# Expression of uncharacterized male germ cell-specific genes and discovery of novel sperm-tail proteins in mice

**DOI:** 10.1371/journal.pone.0182038

**Published:** 2017-07-25

**Authors:** Jun Tae Kwon, Sera Ham, Suyeon Jeon, Youil Kim, Seungmin Oh, Chunghee Cho

**Affiliations:** School of Life Sciences, Gwangju Institute of Science and Technology, Gwangju, Korea; University of Texas at Austin Dell Medical School, UNITED STATES

## Abstract

The identification and characterization of germ cell-specific genes are essential if we hope to comprehensively understand the mechanisms of spermatogenesis and fertilization. Here, we searched the mouse UniGene databases and identified 13 novel genes as being putatively testis-specific or -predominant. Our *in silico* and *in vitro* analyses revealed that the expressions of these genes are testis- and germ cell-specific, and that they are regulated in a stage-specific manner during spermatogenesis. We generated antibodies against the proteins encoded by seven of the genes to facilitate their characterization in male germ cells. Immunoblotting and immunofluorescence analyses revealed that one of these proteins was expressed only in testicular germ cells, three were expressed in both testicular germ cells and testicular sperm, and the remaining three were expressed in sperm of the testicular stages and in mature sperm from the epididymis. Further analysis of the latter three proteins showed that they were all associated with cytoskeletal structures in the sperm flagellum. Among them, MORN5, which is predicted to contain three MORN motifs, is conserved between mouse and human sperm. In conclusion, we herein identify 13 authentic genes with male germ cell-specific expression, and provide comprehensive information about these genes and their encoded products. Our finding will facilitate future investigations into the functional roles of these novel genes in spermatogenesis and sperm functions.

## Introduction

During spermatogenesis, germ cells are processed from primordial germ cells (PGCs) to mature sperm [1, 2]. The tightly regulated nature of this process, which occurs in the seminiferous tubules of testes, indicates that a highly organized network of genes is expressed in male germ cells during their development [3]. An intrinsic program determines which genes are utilized and when they are expressed in germ cells, and an interactive regulation between germ cells and somatic cells is necessary for germ cell proliferation and progression. Extrinsic influences, including steroid and peptide hormones, control this interactive regulation. However, the intrinsic genetic program is central to the development and fertilization of male germ cells.

Many unique genes and variant transcripts are expressed during spermatogenesis, and the identification and characterization of male germ cell-specific genes are crucial to our understanding of the mechanisms of sperm development [4, 5]. Analysis of the proteins encoded by such genes has revealed that they are developmentally regulated and involved in diverse functions during spermatogenesis and fertilization [6–8]. For example, Mm.290718/*Zfp541* was found to encode a protein that forms a complex involved in chromatin remodeling during late spermatogenesis [7]. The protein encoded by Mm.87328/*Shsp1* localizes to the head region of mature sperm, particularly the surface of the acrosomal region [6]. Finally, three novel male germ cell-specific genes (Mm.386907/*Sfap1*, Mm.157049/*Sfap2*, and Mm.442063/*Als2r12*) were found to encode proteins associated with cytoskeletal structures in the sperm flagellum [6, 8].

Here, as part of an ongoing study of germ cell-specific genes, we identified 13 testis-specific genes using sequence information from the mouse testis UniGene database, and analyzed their characteristics at the gene and transcript levels. Furthermore, we obtained original findings on the developmental expression patterns and localizations of seven of the encoded proteins. In particular, we found that three of the novel proteins were present in mature sperm and associated with cytoskeletal structures of the sperm tail. Therefore, our study represents the first transcript- and protein-level characterization of 13 novel testis-specific genes that may play roles in spermatogenesis and fertilization.

## Materials and methods

### Ethics statements

The biospecimens used in this study were provided by the Pusan National University Hospital, which is a member of the National Biobank of Korea supported by the Ministry of Health, Welfare and Family Affairs. All samples from the National Biobank of Korea were obtained with informed consent under institutional review board-approved protocols. The study of human sperm was also ratified through the Ethics Committee of Gwangju Institute of Science and Technology (GIST) and Chonnam National University (permit number: 20140818-BR-14-01-02). All participants signed an informed consent form permitting use of their semen remnants in this study.

### UniGene database analysis

Mouse UniGene profile data (Build #193) was obtained from the NCBI UniGene database. The profile data contained expressed sequence tag (EST) expression profile of each gene entry in 47 different tissues. Transcripts per million (TPM, indicating the normalized expression levels) and testis specificity were calculated as described [9]. Genes with unassigned functions were regarded as 'unknown' according to Gene References into Function (GeneRIF) in the NCBI Gene database. We discarded previously characterized testis-specific genes by PubMed search.

### Reverse-transcription (RT) PCR

RT-PCR experiments were performed using total RNA from eight tissues (testis, brain, heart, kidney, liver, lung, muscle, and spleen) of male mice. RT-PCR was also performed using RNA from germ cell-lacking testes of *W/W*^*v*^ mutant mice (SLC) [10], to determine whether the genes were expressed in somatic cells of the testis. Total RNA was extracted using the TRIzol reagent (Molecular Research Center) according to the manufacturer’s protocol, and cDNA was synthesized by random hexamer and oligo(dT) primers using the Omniscript reverse transcriptase (Qiagen). The utilized gene-specific primers are listed in S1 Table. PCR was performed for 30 cycles of 94°C for 30 s, 55°C for 30 s, and 72°C for 1 min. Glyceraldehyde-3-phosphate dehydrogenase (*Gapdh*) was amplified as a control (forward, 5’-TGA AGG TCG GAG TCA ACG GAT TTG GT-3’ and reverse, 5’-CAT GTG GGC CAT GAG GTC CAC CAC-3’). Specific expression at different stages of spermatogenesis was established using total RNA obtained from the testes of prepubertal and adult male mice (ages 8, 10, 12, 14, 16, 20, and 30 days after birth). All animal investigations were carried out according to the guidelines of the Animal Care and Use of Gwangju Institute of Science and Technology. The protocol was approved by the Animal Care and Use Committee of Gwangju Institute of Science and Technology (Permit number: GIST 2011–13).

### Antibodies

For production of polyclonal antibodies, glutathione S-transferase (GST)-fusion proteins containing the specific antigenic regions of seven candidate proteins were expressed in *Escherichia coli* BL21 and affinity purified with glutathione Sepharose 4B (GE Healthcare). The recombinant proteins were used as antigens for producing rabbit polyclonal antisera. The antibodies were affinity purified using the appropriate proteins and an AminoLink immobilization kit (Pierce). The following commercially available antibodies were also used: a mouse monoclonal antibody against ADAM2 (1/1000, MAB19292) from Millipore; an antibody against α-tubulin (1/1000, T6199) from Sigma-Aldrich; and an antibody against GAPDH (1/1000, MCA4739) from Bio-Rad. As secondary antibodies for Western blot analysis, we used horseradish peroxidase (HRP)-conjugated anti-rabbit or anti-mouse IgG (Jackson ImmunoResearch).

### Preparation of protein samples

Mouse tissues were lysed in a nonionic detergent lysis buffer (1% NP-40) containing 1% protease inhibitor cocktail (GenDEPOT) for 1 h on ice. Debris was removed by centrifugation at 13,000 g for 15 min at 4°C. The supernatant fractions from the lysate were mixed with 2x SDS sample buffer and boiled for 5 min. Testicular cells and sperm were prepared as previously described [11]. Briefly, cells were isolated by suspension in 52% isotonic Percoll (GE Healthcare) followed by centrifugation for 10 min (27,000 g, 4°C), and then resuspended in Mg^2+^-HEPES buffer. Sperm from the cauda epididymis and vas deferens were released into PBS. The sperm suspension was centrifuged twice at 800 g for 3 min to remove contaminants, and the pelleted sperm were resuspended in 2x SDS sample buffer and boiled for 10 min. For sperm fractionation, sperm were collected and sonicated. An equal volume of 1.8 M sucrose was added and the suspension was layered over a discontinuous sucrose gradient containing equal volumes of 2.05 M and 2.2 M sucrose solutions. The sample was centrifuged at 100,000 g at 4°C for 16 h, and the sperm heads and tails were collected from the pellets at the bottom and the middle of the 2.05 M sucrose layer, respectively. To test the solubility of sperm tail proteins, sperm were lysed in lysis buffer (10 mM Tris-Cl, pH 8.0, 150 mM KCl, 5 mM MgCl2, 0.5 mM EDTA) containing 2, 3, 4 or 6 M urea, or in 1% NP-40 or Triton X-100. Lysis was performed on ice for 2 h, except for experiments that used the lysis buffer containing 6 M urea, which involved incubation at room temperature. Soluble and insoluble fractions were separated by centrifugation at 10,000 g for 15 min, and each faction was subjected to Western blot analysis.

### Western blot analysis

Proteins were denatured by boiling for 10 minutes in the presence of 3% SDS and 5% β-mercaptoethanol, separated by SDS-PAGE, and transferred to polyvinylidene difluoride (PVDF) membranes (0.2 μm; PALL Lifesciences). The membranes were blocked with 5% nonfat dry milk in TBS-T (50 mM Tris-HCl, pH 7.5, 150 mM NaCl [TBS] containing 0.1% Tween-20) and incubated with primary antibodies at room temperature for 1 h. After three 10-min washes with TBS-T, the membranes were incubated with HRP-conjugated secondary antibodies for 1 h at room temperature. Following three washes with TBS-T, immunoreactive proteins were detected using a chemiluminescence kit (Thermo Scientific).

### Immunofluorescence

Freshly dissected testes were fixed in 4% paraformaldehyde, embedded in paraffin and sectioned at 5 μm. The sections were deparaffinized in xylene and an ethanol series. Antigen retrieval was performed by boiling the sections for 20 min in 10 mM tribasic sodium citrate, pH 6. The samples were washed in phosphate-buffered saline (PBS) and then immersed in 0.4% Triton X-100 for 10 min before being blocked with a buffer (3% bovine serum albumin in PBS) for 30 min. The sections were incubated overnight at 4°C with the primary antibodies (5 μg·mL^-1^), and then placed in 1/1000 dilutions of Rhodamine Red™-X goat anti-rabbit IgG (Molecular Probes) for 30 min at room temperature. DNA was stained with DAPI, and the sections were mounted and visualized by fluorescence microscopy (DMLB, Leica).

## Results

### Identification of testis-specific novel genes

To select for genes that are exclusively transcribed in testis, we calculated testis specificity using information from the mouse UniGene database (Build #193) [9], which includes the EST expression profile of a given gene in particular tissues of mice at a specific age and/or health status, as expressed in TPM. We selected genes on the basis of the following criteria: (i) genes with testis specificity >50% and (ii) genes with unassigned function. We collected the information available for the selected genes, such as the presence of coding sequences, known homologies, and cell lines with known mutations. Using this strategy, we identified 13 novel genes as being putatively testis-specific or -predominant. The UniGene ID numbers and names of these genes are Mm.276332/MORN repeat containing 5 (*Morn5*); Mm.23509/phosphatidylethanol amine binding protein 4 (*Pebp4*); Mm.56430/coiled-coil glutamate rich protein 1 (*Ccer1*); Mm.73222/testis expressed 33 (*Tex33*); Mm.131623/transmembrane and coiled-coil domains 5 (*Tmco5*); Mm.269049/*1700001P01Rik*; Mm.157047/*4933417A18Rik*; Mm.271255/*1700013F07Rik*; Mm.272519/*1700013G24Rik*; Mm.87624/family with sequence similarity 71, member F1 (*Fam71f1*); Mm.258841 /family with sequence similarity 71, member E1 (*Fam71e1*); Mm.159422/*4930505A04Rik*; and Mm.46148/family with sequence similarity 209 (*Fam209*).

### Expression patterns of the 13 novel genes

**Fig 1:**
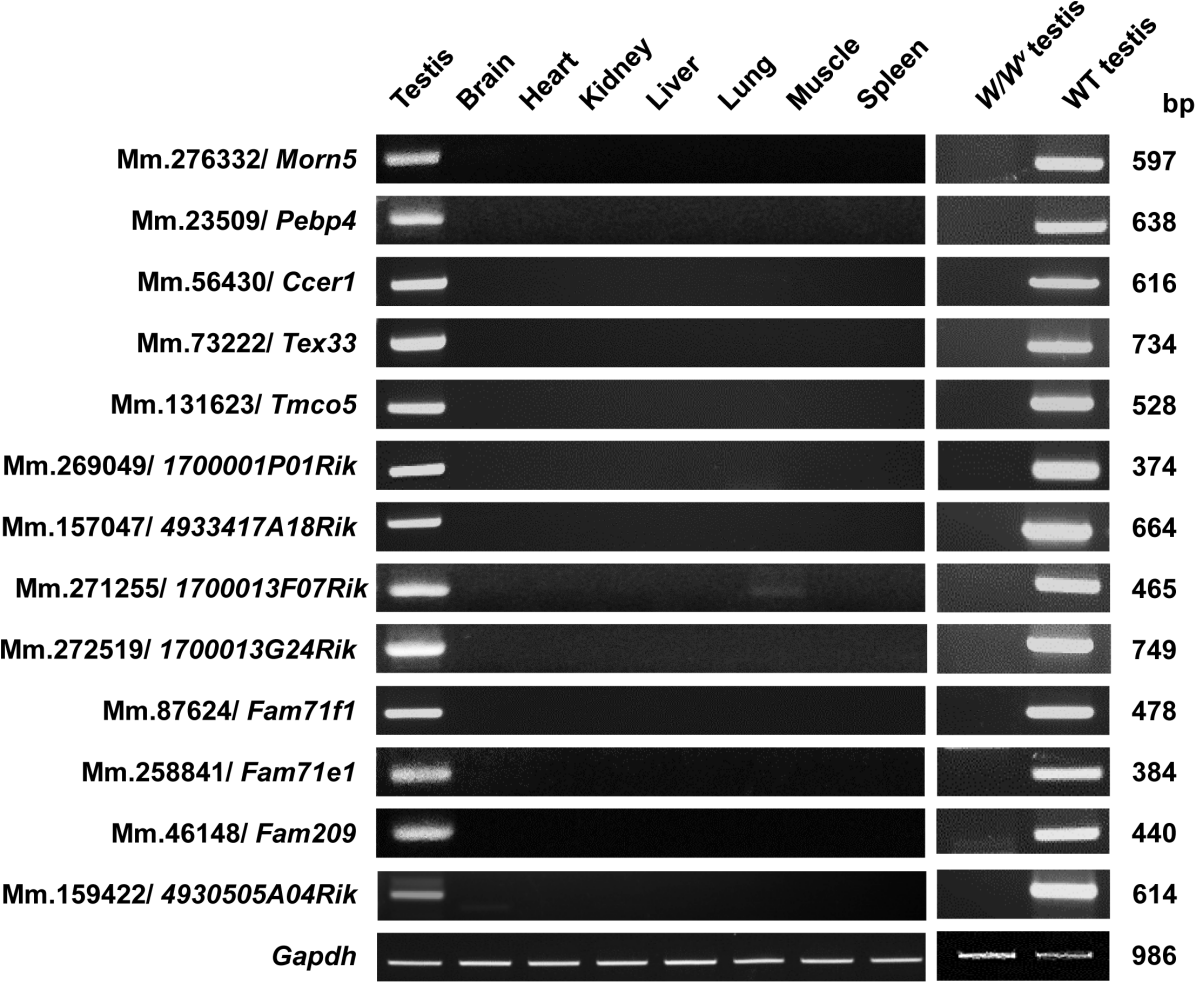
Testis- and germ cell-specific expression of the 13 novel genes. The tissue distributions of the 13 selected genes were assessed by RT-PCR in eight different tissues of adult male mice. Germ cell-specific expression was also examined in germ cell-lacking testes from *W/W*^*v*^ mutant mice. Glyceraldehyde-3-phosphate dehydrogenase (*Gapdh*) was amplified as a loading control. WT, wild-type testis; *W/W*^*v*^, germ cell-less testis. All genes exhibited testis- and germ cell-specific expression.

To determine whether the genes selected from the mouse UniGene library represented authentic genes with testis-specific expression, we performed various expression analyses. RT-PCR analysis showed that all of the genes were expressed at the expected sizes exclusively in mouse testis, which was consistent with our *in silico* prediction (Fig 1). When we examined their expressions in the germ cell-lacking testis of *W/W*^*v*^ (c-kit) mutant mice [10], we found little or no expression of the selected genes (Fig 1), further validating that their expression is germ cell-specific.

**Fig 2:**
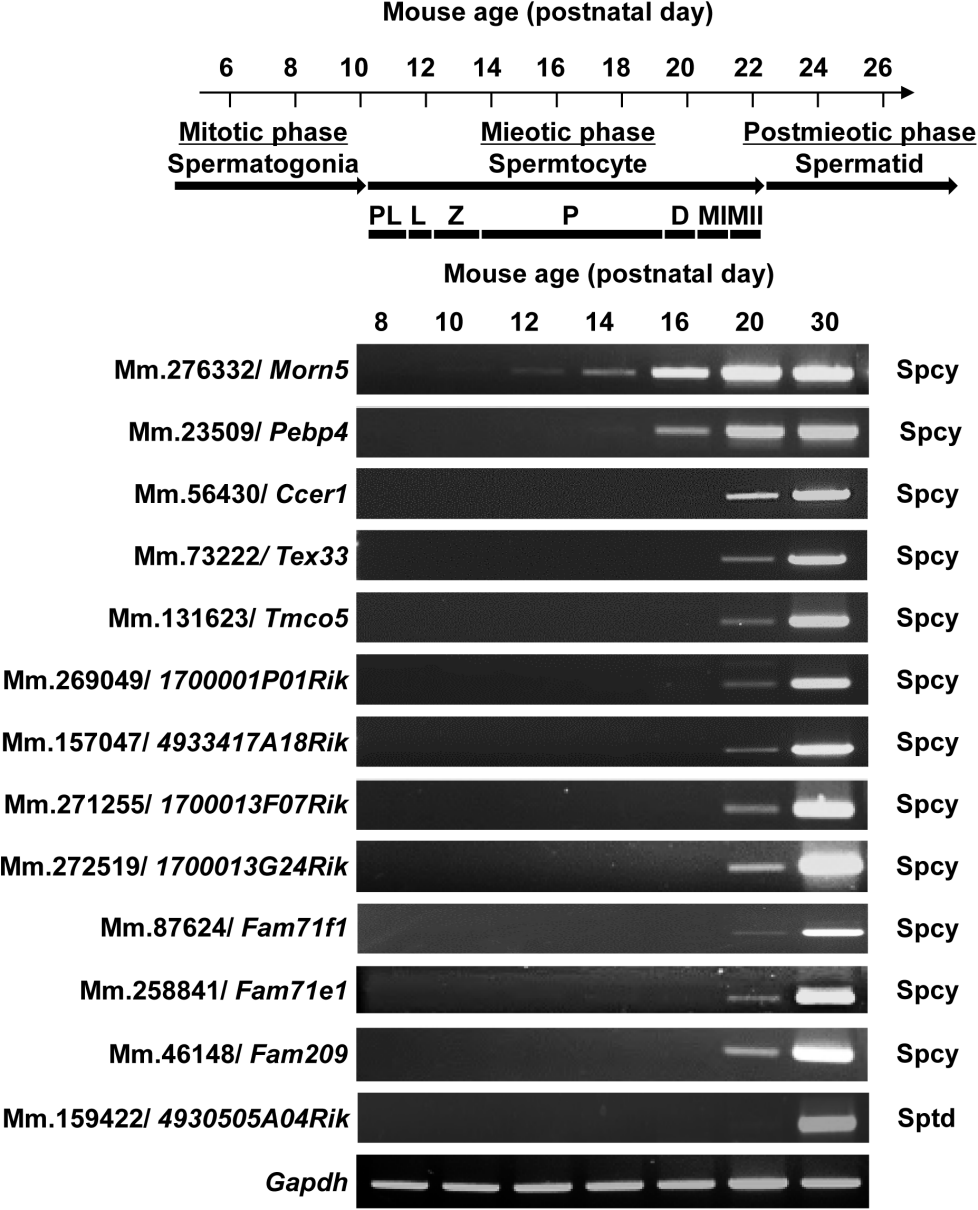
Developmental expression of the selected genes during spermatogenesis. Juvenile spermatogenesis consists of the mitotic, meiotic and postmeiotic phases. The meiotic phase includes the preleptotene (PL), leptotene (L), zygotene (Z), pachytene (P), and diplotene (D) stages. Diplotene spermatocytes undergo two meiotic divisions (MI and MII). The stage-specific expressions of the selected genes were determined from mouse testes obtained on days 8, 10, 12, 14, 16, 20, and 30 after birth. *Gapdh* was included as a loading control. All of the selected genes, with the exception of Mm.159422, were expressed from spermatocytes. Spcy, spermatocytes; Sptd, round spermatids.

In the first round of spermatogenesis in a prepubertal mouse, spermatogonial stem cells increasingly proliferate and differentiate through the sequence of spermatogonia, spermatocytes, and spermatids (Fig 2) [12]. We hypothesized that if a given gene is transcribed in germ cells during spermatogenesis, the transcripts will appear in the testis at an exact post-partum time point corresponding to a specific stage of spermatogenesis. Indeed, our RT-PCR results showed that all of the selected genes were expressed at least after day 12, indicating that their expressions are developmentally regulated. With the exception of Mm.159422/*4930505A04Rik*, the examined genes were all first expressed between day 12 and day 20, which corresponds to the spermatocyte stage. Mm.159422/*4930505A04Rik* was expressed in germ cells after meiosis (Fig 2). Collectively, the results of our *in vitro* analyses indicate that the *in silico*-selected genes show testis- and germ cell-specific expression, and are developmentally regulated during spermatogenesis.

### *In silico* analysis of genomic, transcript, and protein characteristics

**Fig 3:**
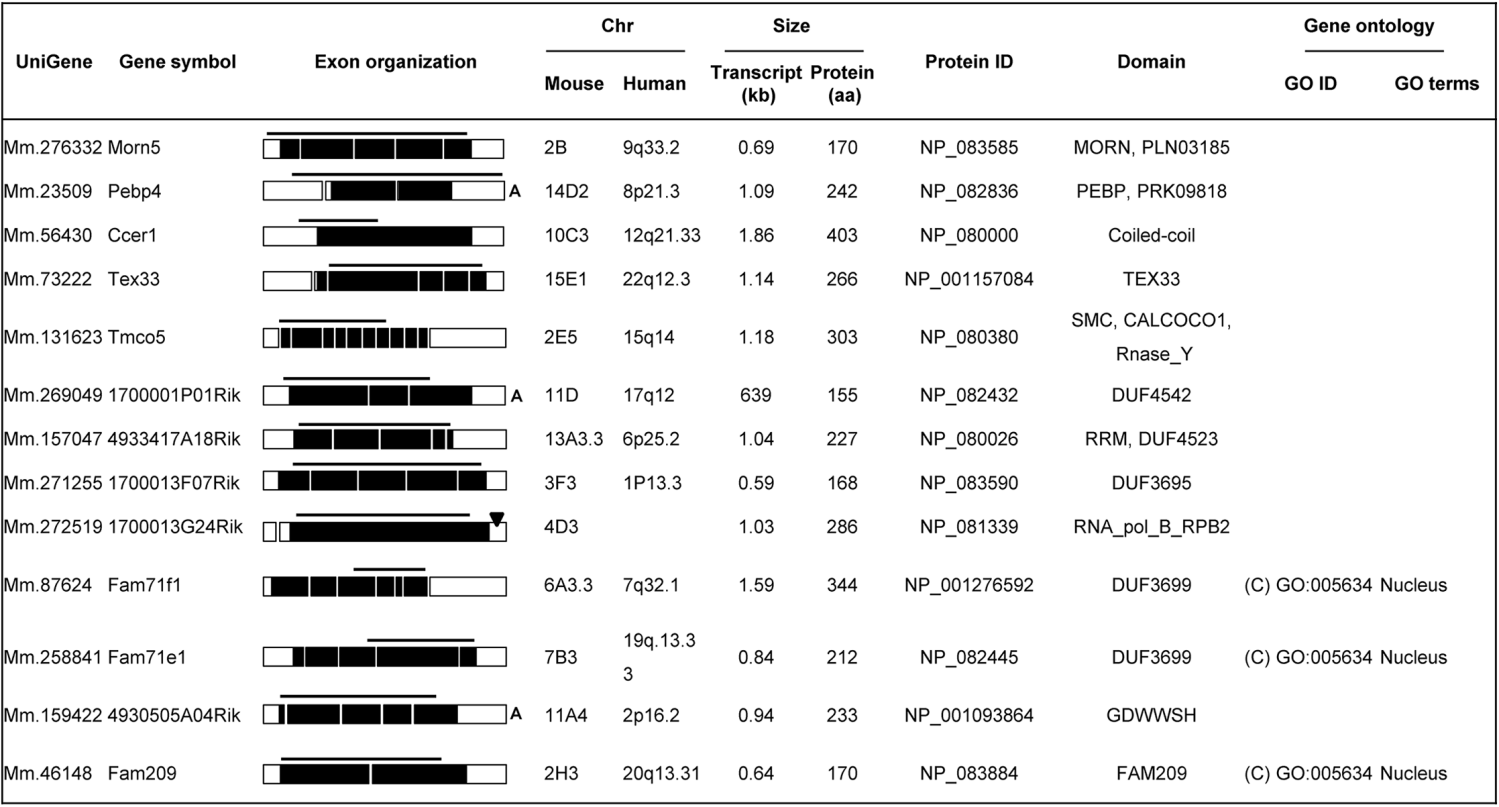
*In silico* analysis of the genomic, transcript, and protein characteristics of the selected genes. The chromosomal localization and intron-exon organization of each gene were determined by genome database searches. Under ‘exon organization,’ the boxes indicate exons. The bars represent the regions amplified in our PCR analysis. The protein-coding regions (black-shaded) were determined by selecting the longest open reading frames deduced from the predicted cDNA sequences. The position of the poly A signal is marked by an arrowhead, and the presence of poly A is indicated by ‘A.’ The Gene Ontology (GO) terms for each gene product were obtained from the Gene Ontology Consortium; they all fall under the broad categories of molecular function (M), cellular component (C), and biological process (B).

To characterize the genomic, transcript, and protein natures of the selected genes, we collected the information available from various databases. Fig 3 shows the exon organizations and chromosomal locations of the genes, the predicted transcript sizes, and the numbers of amino acids, specific domains, and gene ontology (GO) of the predicted proteins. The exon numbers of the genes ranged from 1 to 12 exons, and the genes were found to be widely distributed across the mouse chromosomes. To extend our findings, we searched the human genome database and found that human orthologs for 12 of the 13 selected mouse genes are present in genomic regions that show conserved synteny between mice and humans. Ten of these human genes were predicted to be expressed predominantly in testis; MORN5 and PEBP4 were not. The protein-coding region of each gene was defined by selecting the longest amino acid sequence before a stop codon. All of the translated gene products were predicted to contain various domains and motifs, and could be annotated with GO codes.

### Generation of antibodies and expression patterns of novel proteins

To further investigate the characteristics of the proteins encoded by the selected genes, we generated antibodies against seven of them: Mm.276332/MORN5, Mm.258841/FAM71E1, Mm.272519/1700013G24Rik, Mm.73222/TEX33, Mm.131623/TMCO5, Mm.159422/4930505 A04Rik and Mm.271255/1700013F07Rik. GST-fused recombinant proteins were produced and used to generate polyclonal sera (S1 Fig), and affinity purification of antibodies was performed against GST and GST fusion proteins. To verify the specificity of the antibodies for the novel proteins and confirm whether the proteins were expressed in testis, we performed Western blot analysis with lysates of liver and testis. All of the generated antibodies recognized appropriately sized bands in the testis, but not in the liver, and the bands disappeared when the GST-fusion proteins were added along with the primary antibodies (S2 Fig). These results support the specificity of the antibodies and the authenticity of the selected proteins.

**Fig 4:**
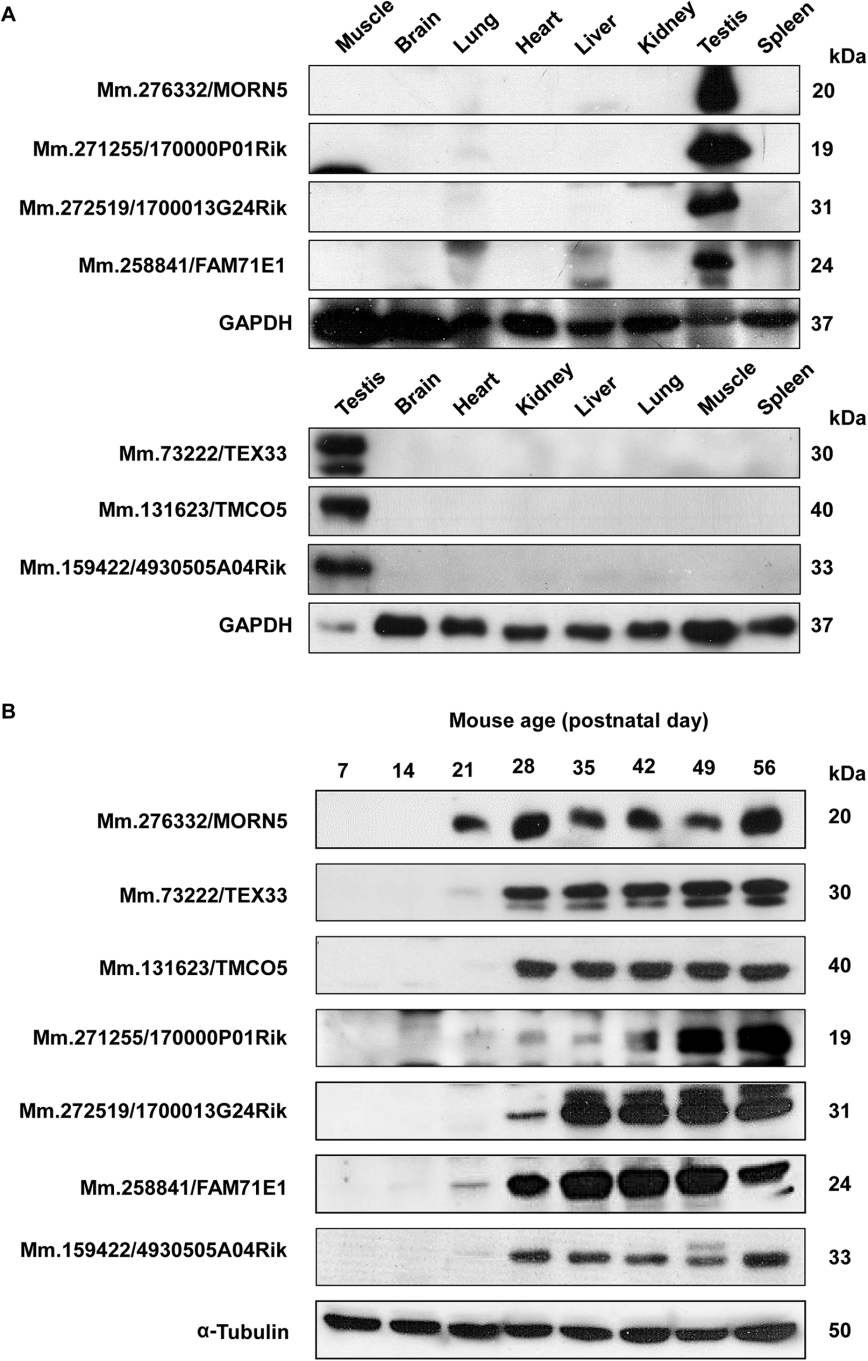
Expression patterns of seven of the novel proteins in various tissues and the postnatal testes of mice. A. The tissue distributions of some of the novel proteins were examined by immunoblotting, with GAPDH detected as a loading control. All of the gene products showed specific expression in testis. B. The stage-specific expressions of the novel proteins were determined from mouse testes obtained on days 7, 14, 21, 28, 35, 42, 49, and 56 after birth. The anti-α-tubulin antibody was used as a control. All of the proteins were expressed in late meiotic or postmeiotic germ cells.

To determine whether the selected proteins are expressed exclusively in testis, we performed Western blot analysis with protein extracts from eight different tissues. Consistent with our RT-PCR results, all of the proteins were expressed specifically in the testis (Fig 4A and S3 and S4 Figs). Further immunoblot analyses performed with mouse testes obtained at different times after birth revealed that Mm.276332/MORN5 and Mm.258841/FAM71E1 were expressed in spermatocytes (day 21); Mm.271255/1700013F07Rik was weakly expressed in spermatocytes during the first spermatogenesis; and Mm.73222/TEX33, Mm.131623/TMCO5, Mm.272519/1700013G24Rik, and Mm.159422/4930505A04Rik were expressed in round spermatids (day 28) (Fig 4B and S5 Fig).

**Fig 5:**
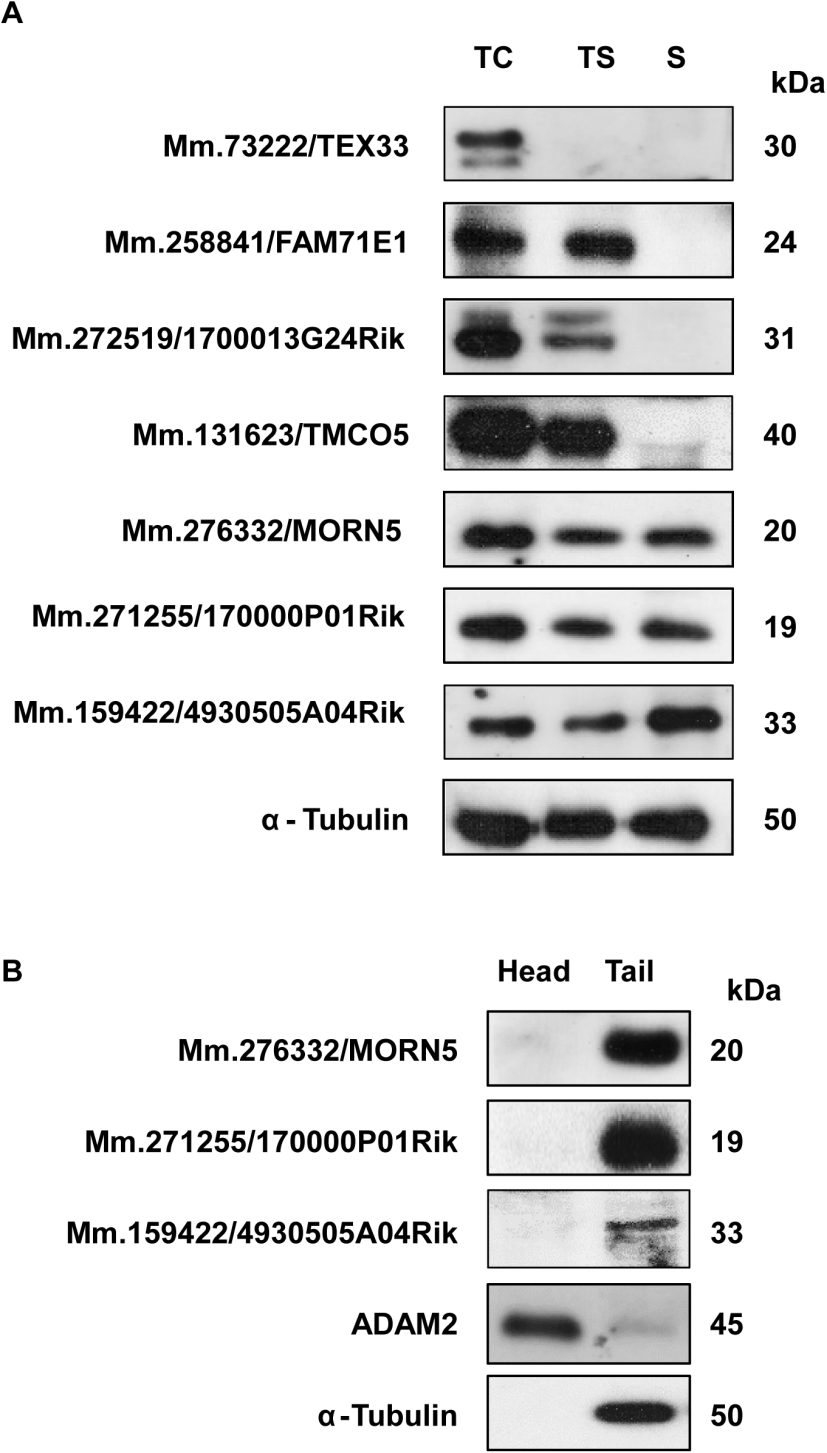
Developmental expression pattern of the novel proteins. A. Protein samples from testicular cells (TC), testicular sperm (TS), and mature sperm (S) were blotted with antibodies against the novel proteins. α-Tubulin was detected as a control. The expressions of the selected proteins were found to be developmentally regulated during sperm maturation. B. Sperm from the cauda epididymis and vas deferens were separated into head (H) and tail (T) fractions, and subjected to Western blot analysis. ADAM2 and α-tubulin were used to confirm the head and tail fractions, respectively. All of the selected proteins were found to be expressed in the sperm tail.

To investigate the expression pattern of the selected proteins in germ cells during sperm development, we performed immunoblotting with lysates of testicular cells (TC), testicular sperm (TS), and mature sperm (S) from the cauda epididymis and vas deferens (Fig 5A and S6 Fig). We found that Mm.73222/TEX33 was present only in testicular cells; Mm.258841/FAM71E1, Mm.131623/TMCO5, and Mm.272519/1700013G24Rik existed in testicular cells and sperm but not in mature sperm; and Mm.276332/MORN5, Mm.271255/1700013F07Rik, and Mm.159422/4930505A04Rik were present in all stages. None of these proteins exhibited any change in molecular weight during spermatogenesis. These results indicate that the seven analyzed proteins exhibited differential regulation during male germ cell development.

**Fig 6:**
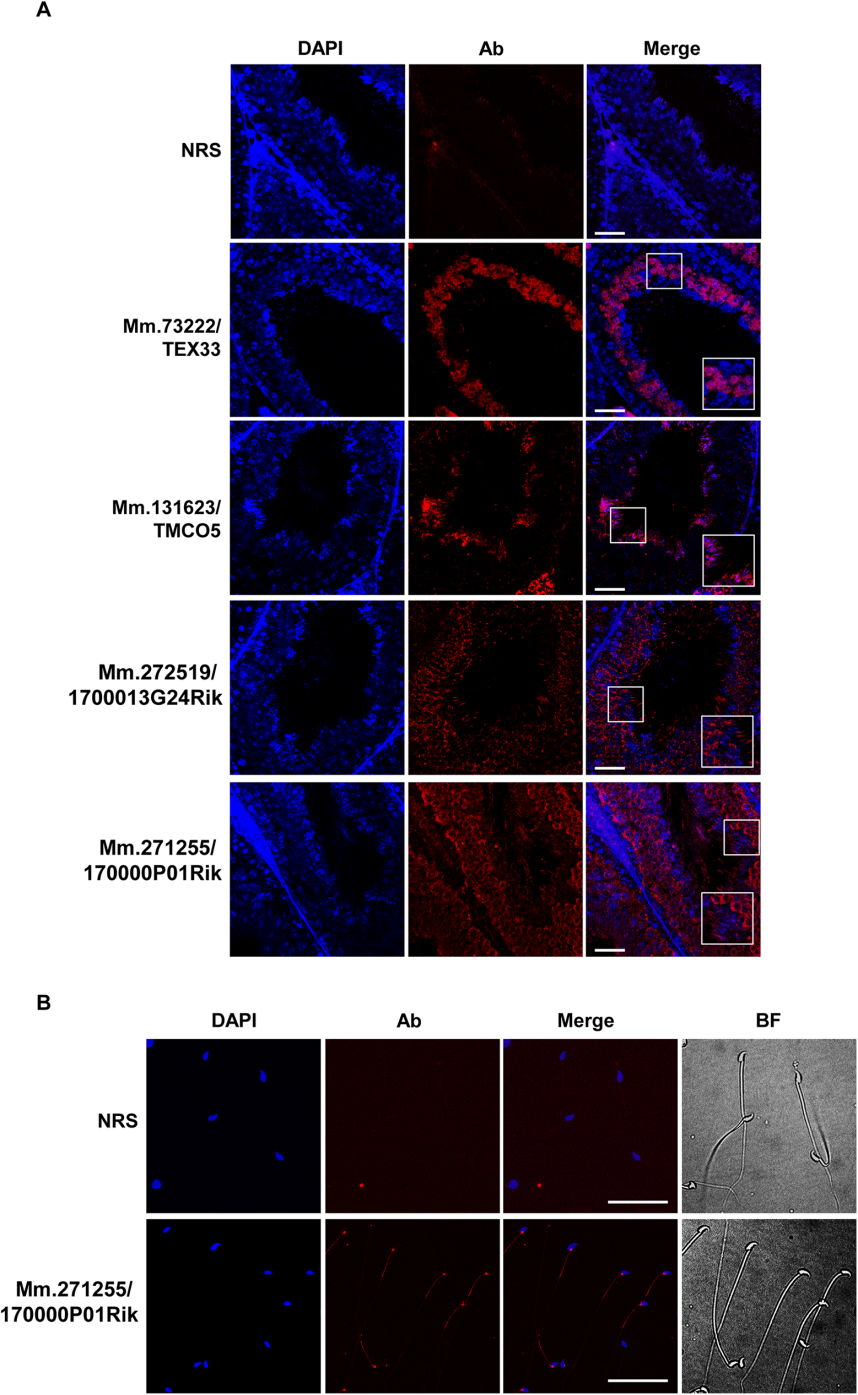
Localization of the novel proteins in adult testis. A. Immunofluorescence staining of paraffin sections of adult testis was conducted using specific antibodies for the four proteins. Normal rabbit serum (NRS) was used as a negative control. The red color indicates proteins and nuclei were stained with DAPI (blue). Images in the boxes are magnified in the insets in merged images. Scale bar, 100 μm. B. Localization of Mm.271255/1700013F07Rik in mature sperm. Sperm from the cauda epididymis and vas deferens were immunostained with anti-Mm.271255/1700013F07Rik. DAPI was used to stain nuclei. Mm.271255/1700013F07Rik is localized to the neck and midpiece of sperm. Scale bar, 100 μm.

To confirm and further investigate the developmental expression and localization of the selected proteins, we performed immunofluorescence analysis in paraffin sections of adult testis. Antibodies to Mm.276332/MORN5, Mm.258841/FAM71E1, and Mm.159422/4930505A04Rik did not display immunoreactivity (S8 Fig). In contrast, we observed immunofluorescence signals in analyses with antibodies to the other proteins (Fig 6A). Mm.73222/TEX33 was found to exist in the cytoplasm of round spermatids. Mm.131623/TMCO5 was identified exclusively in the tail region of elongated spermatids. Mm.272519/1700013G24Rik showed a dot pattern in the cytoplasm of round spermatids, but was mainly present in the tail of elongated spermatids. Mm.271255/1700013F07Rik was expressed in the cytoplasm of spermatocytes and round spermatids, and then observed in the tail of elongated spermatids (Fig 6A). These data confirm and extend the Western blot results (Figs 4 and 5).

### Localization and characterization of novel proteins in mature sperm

Because three of the novel proteins (Mm.276332/MORN5, Mm.271255/1700013F07Rik, and Mm.159422/4930505A04Rik) were found to exist in mature sperm, we hypothesized that they might be related to sperm function and fertilization. To elucidate the distribution of these proteins in mature sperm, we isolated sperm from the epididymis and vas deferens, separated them into head and tail fractions, and subjected the protein lysates of these fractions to Western blot analysis. ADAM2 and α-tubulin were used as positive controls for the head and tail fractions, respectively. All three proteins were found exclusively in the tail fraction (Fig 5B and S7 Fig). In addition, immunofluorescence analysis revealed that Mm.271255/1700013F07Rik was localized to the neck and midpiece of sperm tail (Fig 6B).

**Fig 7:**
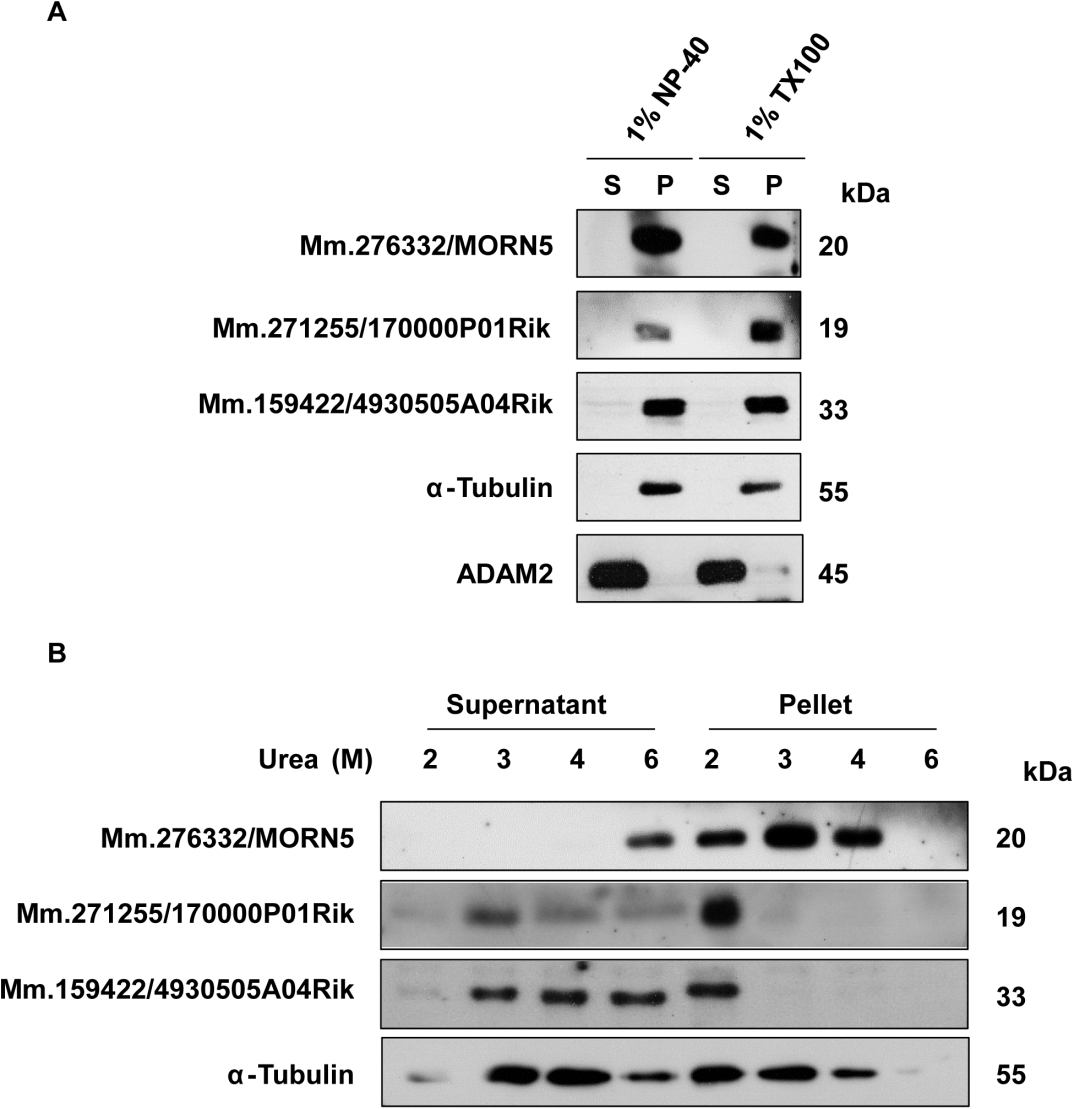
Characterization of three sperm tail proteins. A. Sperm from the epididymis and vas deferens were treated with 1% NP-40 or 1% Triton X-100 and then centrifuged. Soluble and insoluble fractions were subjected to immunoblot analysis. ADAM2 and α-tubulin were used to verify the soluble and insoluble fractions, respectively. The tested proteins failed to solubilize with these detergents. S, supernatant after centrifugation; P, pellet after centrifugation. B. Sperm were treated with 2, 3, 4, or 6 M urea and then centrifuged. Soluble and insoluble fractions were subjected to Western blot analysis, with α-tubulin detected as a loading control. MORN5 and Mm.271255 were found to be insoluble in urea.

Outer dense fibers (ODFs) and the fibrous sheath (FS) are specialized cytoskeletal structures of the mammalian sperm tail. The proteins that comprise these structures show similar solubilities in various detergents [13]. To examine the possible association of Mm.276332/MORN5, Mm.271255/1700013F07Rik, and Mm.159422/4930505A04Rik with these structures, we examined their solubilities in nonionic detergents and 2–6 M urea. None of the tested proteins was solubilized by 1% NP-40 or 1% Triton X-100 (Fig 7A and S9 Fig). Mm.276332/MORN5 was solubilized only in 6 M urea, Mm.271255/1700013F07Rik and Mm.159422/4930505A04Rik resisted the lower concentrations but showed a gradual increase in solubility as the concentration of urea increased (Fig 7B and S9 Fig). These results indicate that MORN5, Mm.271255, and Mm.159422 are likely to be components of or associated with cytoskeletal structures of the sperm flagellum, such as ODFs and the FS.

### Expression of MORN5 in human sperm

**Fig 8:**
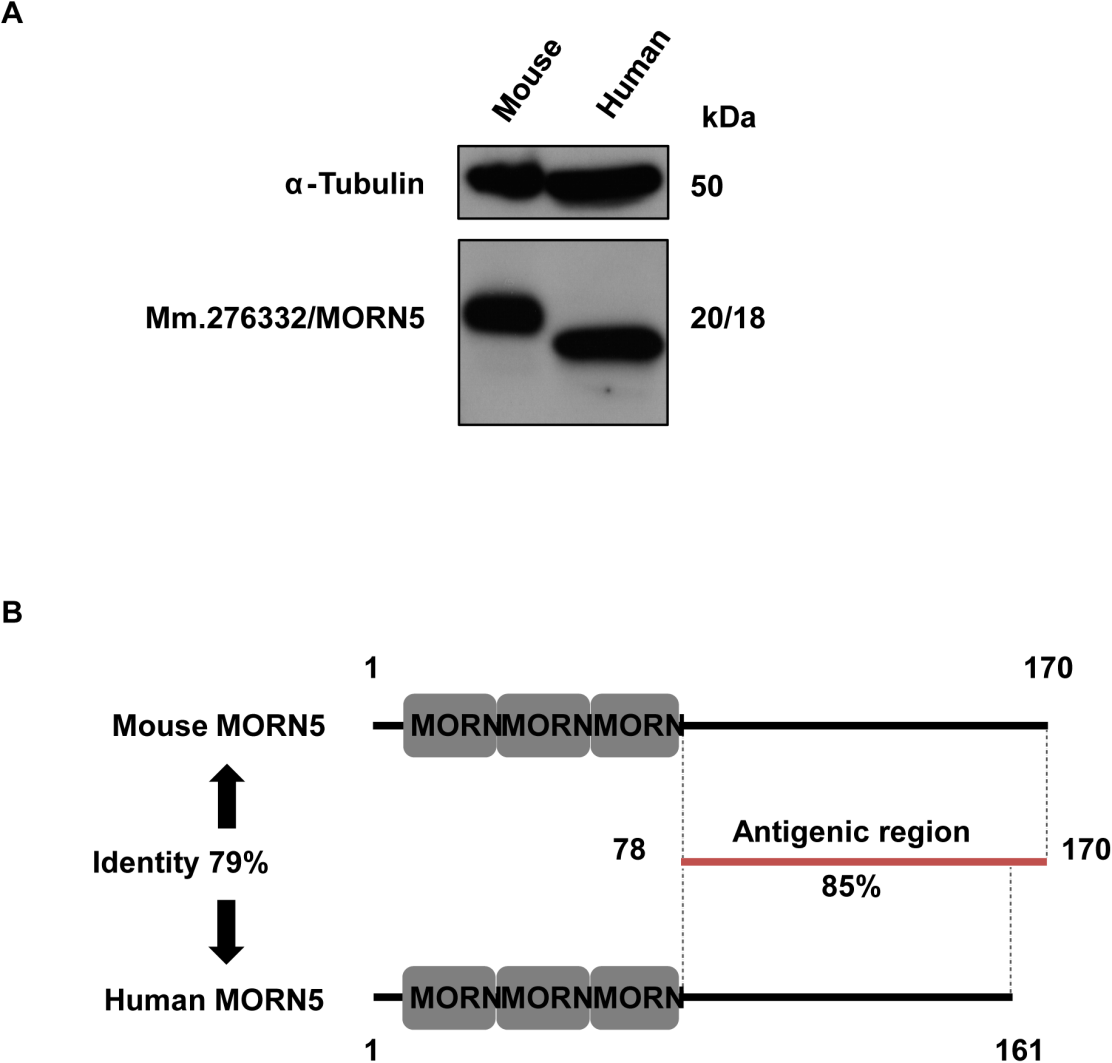
Expression of MORN5 in human sperm. A. Extracts of sperm from mouse and humans were subjected to SDS-PAGE and blotted with anti-MORN5. Tubulin was detected as a loading control. MORN5 was expressed in human sperm. B. Diagram of human and mouse MORN5, showing amino acid identities and domain information. The predicted MORN5 proteins are 170 and 161 amino acids in mouse and human, respectively, and contain three MORN motifs (gray boxes). The antigenic region used to raise the mouse MORN5 antibody is indicated by a red line. This region showed 85% identity between the mouse and human protein sequences (whole protein: 79% identity).

As the three sperm tail proteins (Mm.276332/MORN5, Mm.271255/1700013F07Rik, and Mm.159422/4930505A04Rik) identified from the mouse database showed 68–79% sequence identity to their human homologs, we hypothesized that our generated antibodies might recognize the human homologs. To examine this possibility, we performed Western blot analysis with protein extracts from mouse and human sperm. As shown in Fig 8A, the anti-MORN5 antibody recognized an 18-kDa band in human sperm, which was 2-kDa smaller than that recognized in mouse sperm. The antigenic region of MORN5 exhibits an 85% sequence identity between mouse and human (Fig 8B). These findings suggest that MORN5 might be functionally conserved in mouse and human sperm. Our Western blot analyses using the antibodies against Mm.271255/1700013F07Rik and Mm.159422/4930505A04Rik did not recognize any band with the expected size of the proteins in human sperm (S10 Fig), suggesting that these mouse antibodies did not cross-react with their human orthologs.

## Discussion

In our previous studies, we identified a number of novel male germ cell-specific genes from the McCarrey Eddy spermatocyte (Lib. 6787) and spermatid (Lib.6786) UniGene libraries [4,5]. These two libraries contain 3513 putative gene entries, including ubiquitously expressed genes. Thus, it is possible that a number of spermatogenic cell-specific genes are absent from these data sets. In the present study, we analyzed all mouse testis UniGene libraries and identified 13 authentic genes as being putatively testis-specific or -predominant. Our RT-PCR analysis confirmed that all of the selected genes are testis- and germ cell-specific. Consistent with previous findings that male germ cell-specific genes characteristically show developmental regulation during the meiotic and postmeiotic phases [1, 2], the expression patterns observed for the 13 selected genes during postnatal testicular development were indicative of developmental regulation. With the exception of Mm.159422/4930505A04Rik, all of the genes were found to be expressed from pachytene and diplotene spermatocytes. We integrated the available *in silico* information on the genomic characteristics and encoded transcripts of the 13 selected genes. We identified human orthologs for 12 of the mouse genes, all of which are found in regions of conserved synteny. With the exception of Mm.276332/*Morn5* and Mm.23509/*Pebp4*, all of the human orthologs were predicted to be testis-specific or -predominant in the human UniGene database. We thus propose that the functions of these genes may be conserved between human and mouse. The proteins encoded by the 13 selected genes all possess conserved domains with unknown function. The MORN5 protein is known to be expressed in chick craniofacial structures and has been shown to be involved in bone morphogenetic protein (BMP) signaling [14]. PEBP4 is a secreted and glycosylated protein that appears to be required for Protein kinase B activation [15]. Thus, MORN5 and PEBP4 may function in signaling pathways during the meiotic and postmeiotic phases. The proteins encoded by Mm.87624/*Fam71f1*, Mm.258841/*Fam71e1*, and Mm.46148/*Fam209* are predicted to localize in the nucleus, and a previous LC-MS/MS study identified the human homologs of FAM71A and FAM71B in the human sperm nucleus [16].

We corroborated the authenticity of seven of the selected genes at the protein level, and further investigated the encoded proteins. Specific antibodies generated against the novel proteins clearly recognized distinct bands of the expected sizes in the testis. Immunoblotting confirmed that the seven proteins were testis-specific. Our analysis of the stage-specific expression of the novel proteins in the postnatal testes of mice revealed that Mm.276332/MORN5, Mm.258841/FAM71E1 and Mm.271255/1700013F07Rik were expressed in spermatocytes (day 21), while Mm.272519/1700013G24Rik, Mm.73222/TEX33, Mm.131623/TMCO5 and Mm.159422/ 4930505A04Rik were first expressed in spermatids (day 28). When we investigated the developmental distribution of the novel proteins during spermatogenesis, we found that Mm.73222/TEX33 was detected only in testicular germ cells; Mm.258841/FAM71E1, Mm.131623/TMCO5, and Mm.272519/1700013G24Rik were present in testicular cells and testicular sperm; and Mm.276332/MORN5, Mm.271255/1700013F07Rik, and Mm.159422/4930505A04Rik were observed during all phases of sperm development and maturation. Further immunofluorescence analysis of four proteins (Mm.73222/TEX33, Mm.131623/TMCO5, Mm.272519/1700013G24Rik, and Mm.271255/1700013F07Rik) confirmed the developmental expression pattern and showed the specific cellular localization of the proteins in spermatogenic cells.

The most remarkable finding of the present work was our discovery that three of the novel proteins, Mm.276332/MORN5, Mm.271255/1700013F07Rik, and Mm.159422/4930505A04Rik, were restricted to the tail region of mature sperm. Notably, Mm.271255/1700013F07Rik was observed to exist at the neck and midpiece regions of sperm tail. The flagellum of a mammalian sperm is divided into the middle, principal, and end pieces. The central core of the flagellum consists of a cytoskeletal structure called the axoneme, and the region between the axoneme and the plasma membrane harbors accessory structures, such as mitochondria, ODFs and the FS. The latter two structures are believed to stiffen the sperm tail while allowing elastic bending. The proteins that comprise ODFs and FS commonly resist non-ionic detergents, and FS proteins are insoluble in 6 M urea [13]. Our Western blot analysis showed that our three identified sperm tail proteins were insoluble in the non-ionic detergents, NP-40 and Triton X-100. Mm.276332/MORN5 was solubilized only in 6 M urea. Mm.271255/1700013F07Rik and Mm.159422/4930505A04Rik gradually solubilized as the concentration of urea increased. These results suggest that Mm.276332/MORN5 is intrinsic component of the FS, while Mm.271255/1700013F07Rik and Mm.159422/4930505A04Rik are ODF components or are weakly associated with the FS.

Finally, we found that the antibody against mouse Mm.276332/MORN5 recognized a protein band corresponding to the expected size of MORN5 in human sperm, suggesting that this protein might be functionally conserved in mouse and human sperm. MORN5 contains three MORN (Membrane Occupation and Recognition Nexus) motifs, which contribute to the plasma membrane-binding capacity of junctophilin type 1 [17]. MORN1 may function as a linker protein between certain membrane regions and cytoskeleton of the parasites such as *Toxoplasma gondii* and other *Apicomplexa* [18]. MOPT and MORN3 were found to be expressed in the acrosomal region of elongating spermatids [19, 20], and a recent study suggested that MORN5 is both regulated by and required for BMP signaling during craniofacial development in chicken [14]. Based on these previous findings, we speculate that MORN5 may function in signaling during sperm development, and that it may participate in the tight interaction that occurs between the plasma membrane and cytoskeletal structures of a mature sperm flagellum.

More than a hundred proteins have been identified in mature sperm tail (S2 Table). Our summary of the results from localization and functional studies of these sperm proteins (Table 1) reveals that 73 of the 111 were restricted to the tail region of mature sperm, and more than half of these tail proteins showed region-specific distributions thought to reflect their molecular functions in the sperm tail. GO analysis showed that these sperm tail proteins participated in various processes and structures, including voltage-gated calcium channel activity, cilium, sperm axoneme assembly, transmembrane transport, sperm capacitation, and sperm motility (S3 Table). Notably, only 21 of these proteins have been characterized in terms of their functions in tail formation and fertilization. For example, ODF1 [21], ODF2 [22], meiosis-specific nuclear structural protein 1 (MNS1) [23], and solute carrier family 22 member 14 (SLC22A14) [24] are reportedly related with sperm structure, while the cation channel sperm associated (CatSper) proteins [25, 26], plasma membrane Ca^2+^-ATPase 4 (PMCA4) [27], and rhophilin associated tail protein 1 (ROPN1) [28] function in sperm motility.

**Table 1:** Characteristics of 111 previously identified proteins of mature sperm tail.

Parameter		Number of proteins
Pattern in sperm	Tail	74
	Head and tail	4
	Head (e,p) and tail	7
	Head (e) and tail	4
	Head (p) and tail	6
	Acrosome and tail	16
Pattern in tail	Whole tail	46
	Mid-piece	18
	Mid-piece and principal piece	15
	Principal piece	21
	Principal piece and end-piece	7
	ND	4
KO mice	Infertile	17
	Subfertile	3
	Fertile	1
	ND	90

e, equatorial region; p, post-equatorial; ND, not determined

In this study, we identified and characterized authentic genes specifically expressed in male germ cells through integrative analyses including genomic, transcript, and protein approaches. In particular, the three sperm-tail proteins are noteworthy because of their potential functions in mature sperm. However, the precise functions of the proteins are currently unknown. Future studies are needed to investigate the functions of our identified tail proteins and their relationships with the other known proteins. This should provide new insights into the role of various proteins in sperm development, motility, and fertilization.

## Supporting information

S1 Fig Protein characteristics of seven candidates. The GenBank accession numbers of the cDNA sequences predicted for the novel genes are listed. The numbers of amino acids, hydrophobicities, and expected molecular weights were predicted from the deduced coding regions of these cDNA sequences. The bars indicate regions corresponding to the antigens used for antibody generation. No, number; AA, amino acid; MW, molecular weight.
(TIF)

S2 Fig Specificity of antibodies to the novel proteins. Total protein lysates were obtained from liver (L) and testis (T) using lysis buffer containing 1% SDS. These samples were subjected to SDS-PAGE under reducing conditions followed by Western blotting with the generated antibodies. All antibodies detected bands of the expected sizes except for the anti-TMCO5 antibody, which recognized a 40-kDa band that was larger than expected. When GST or GST-fusion (GST-F) proteins were mixed with the primary antibodies for immunoblotting, all of the bands disappeared in experiments run with the GST-fused antigens.
(TIF)

S3 Fig Original blots for tissue distributions of Mm.276332/MORN5, Mm.271255/1700013F07Rik, Mm.28841/FAM71E1, and Mm.272519/1700013G24Rik. These are original uncropped and unadjusted blots of four proteins in Fig 4A. Bands corresponding to the proteins are indicated by arrowheads.
(TIF)

S4 Fig Original blots for tissue distributions of Mm.73222/TEX33, Mm.131623/TMCO5, Mm.159422/4930505A04Rik. These are original uncropped and unadjusted blots of three proteins in Fig 4A. Bands corresponding to the proteins are indicated by arrowheads.
(TIF)

S5 Fig Original blots of stage-specific expression pattern. These are original uncropped and unadjusted blots of the proteins in Fig 4B. Bands corresponding to the proteins are indicated by arrowheads.
(TIF)

S6 Fig Original blots of developmental expression pattern. These are original uncropped and unadjusted blots of the proteins in Fig 5A. Bands corresponding to the proteins are indicated by arrowheads.
(TIF)

S7 Fig Original blots of sperm head and tail. These are original uncropped and unadjusted blots of the proteins in Fig 5B. Bands corresponding to the proteins are indicated by arrowheads.
(TIF)

S8 Fig Immunostaining of Mm.276332/MORN5, Mm.258841/FAM71E1, and Mm.159422/4930505A04Rik. Immunofluorescence staining of paraffin sections of adult testis was conducted using specific antibodies to Mm.276332/MORN5, Mm.258841/FAM71E1, and Mm.159422/4930505A04Rik. Nuclei was stained with DAPI (blue). These antibodies did not display immunoreactivity. Scale bar, 100 μm.
(TIF)

S9 Fig Original blots of the solubility of three tail proteins. These are original uncropped and unadjusted blots of the proteins in Fig 7. Bands corresponding to the proteins are indicated by arrowheads.
(TIF)

S10 Fig Expression of Mm.276332/MORN5, Mm.271255/ 1700013F07Rik, and Mm.159422/4930505A04Rik in human sperm. Extracts of sperm from mouse and humans were subjected to SDS-PAGE and blotted. Tubulin was detected as a loading control. MORN5 was expressed in human sperm (A). Mm.271255 (B) and Mm.159422 (C) antibodies did not cross-react with human orthologous proteins. Bands corresponding to the proteins are indicated by arrowheads.
(TIF)

S1 Table Sequences of primers. (DOCX)

S2 Table List of 111 proteins previously identified in mature sperm tail. (DOCX)

S3 Table Gene ontology terms related to sperm tail proteins. (DOCX)
