# Supplementary figures and images for: Expression of uncharacterized male germ cell-specific genes and discovery of novel sperm-tail proteins in mice
Source: PLoS One. 2017 Jul 25;12(7):e0182038. doi: 10.1371/journal.pone.0182038 (PMC5526581; doi:10.1371/journal.pone.0182038)

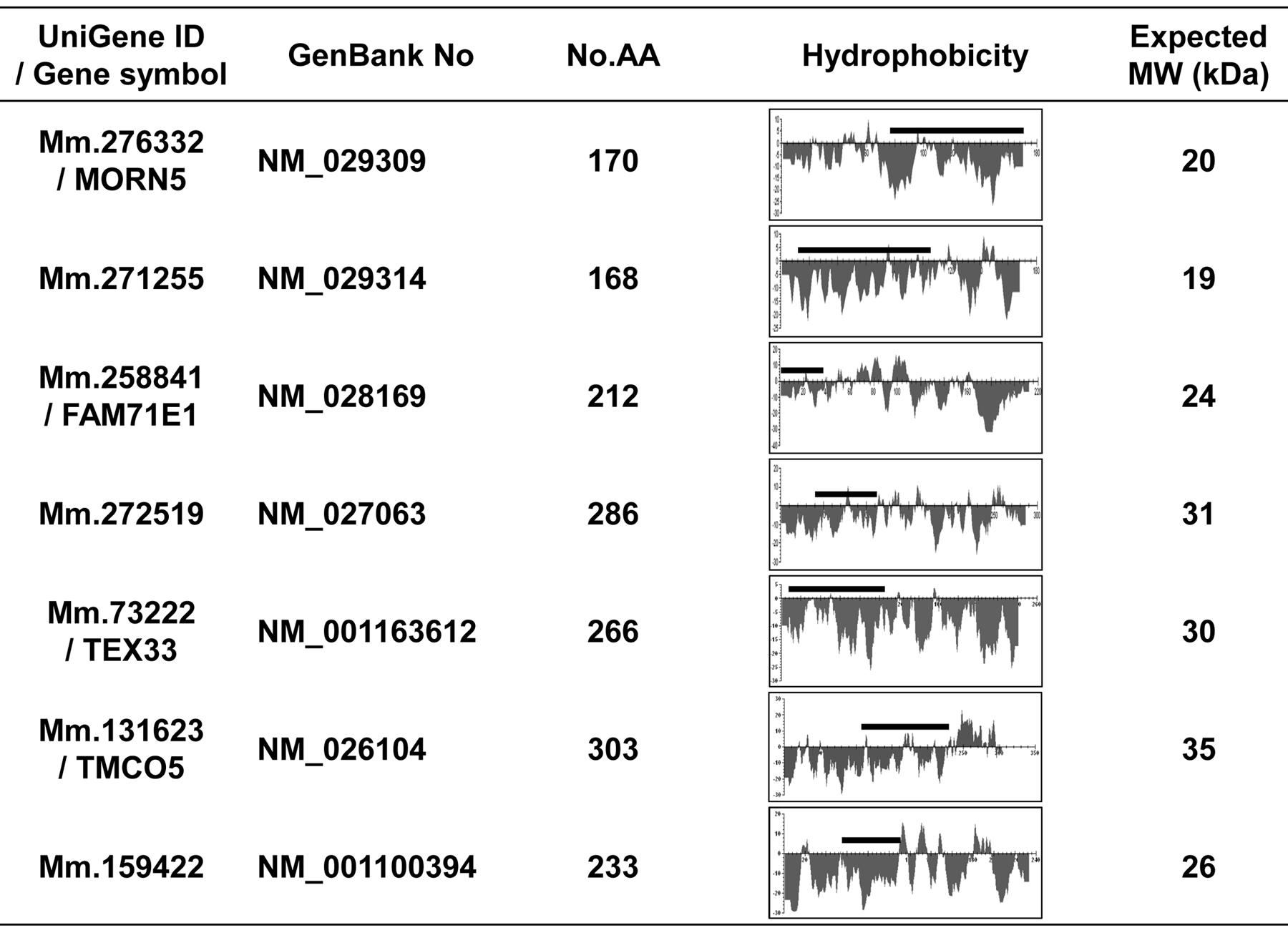

Supplement: S1 Fig — The GenBank accession numbers of the cDNA sequences predicted for the novel genes are listed. The numbers of amino acids, hydrophobicities, and expected molecular weights were predicted from the deduced coding regions of these cDNA sequences. The bars indicate regions corresponding to the antigens used for antibody generation. No, number; AA, amino acid; MW, molecular weight. (TIF) [file pone.0182038.s001.tif]

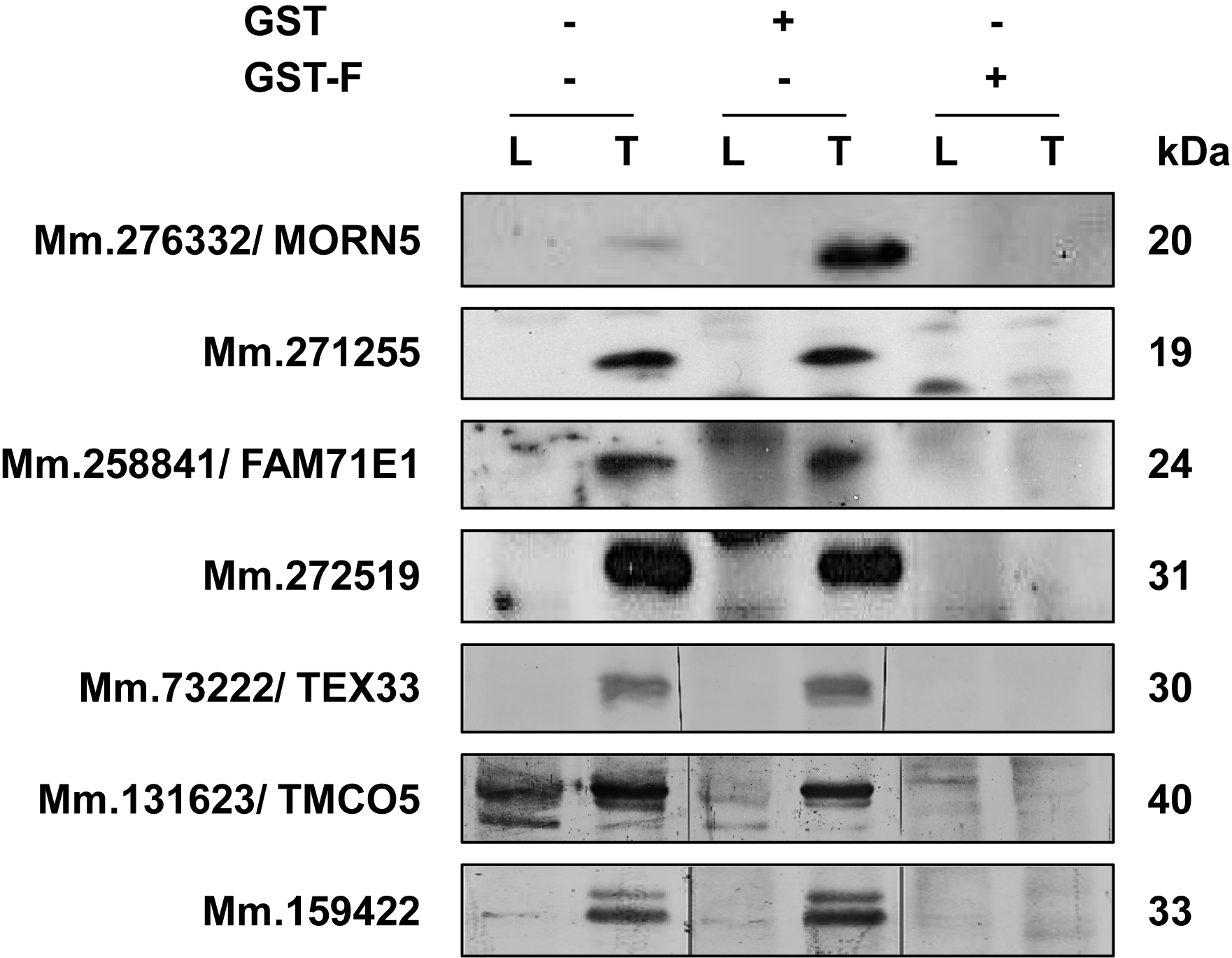

Supplement: S2 Fig — Total protein lysates were obtained from liver (L) and testis (T) using lysis buffer containing 1% SDS. These samples were subjected to SDS-PAGE under reducing conditions followed by Western blotting with the generated antibodies. All antibodies detected bands of the expected sizes except for the anti-TMCO5 antibody, which recognized a 40-kDa band that was larger than expected. When GST or GST-fusion (GST-F) proteins were mixed with the primary antibodies for immunoblotting, all of the bands disappeared in experiments run with the GST-fused antigens. (TIF) [file pone.0182038.s002.tif]

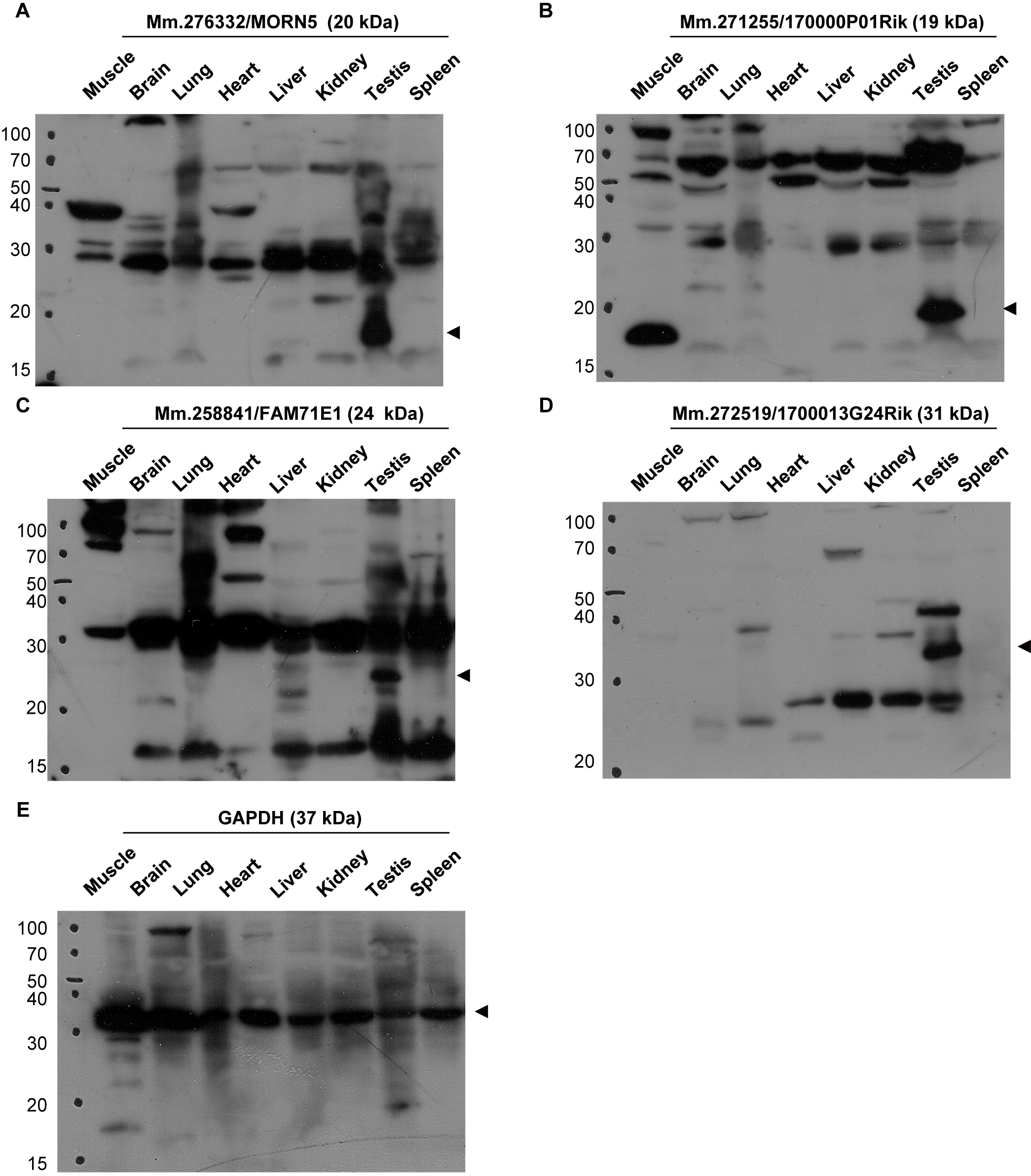

Supplement: S3 Fig — These are original uncropped and unadjusted blots of four proteins in Fig 4A. Bands corresponding to the proteins are indicated by arrowheads. (TIF) [file pone.0182038.s003.tif]

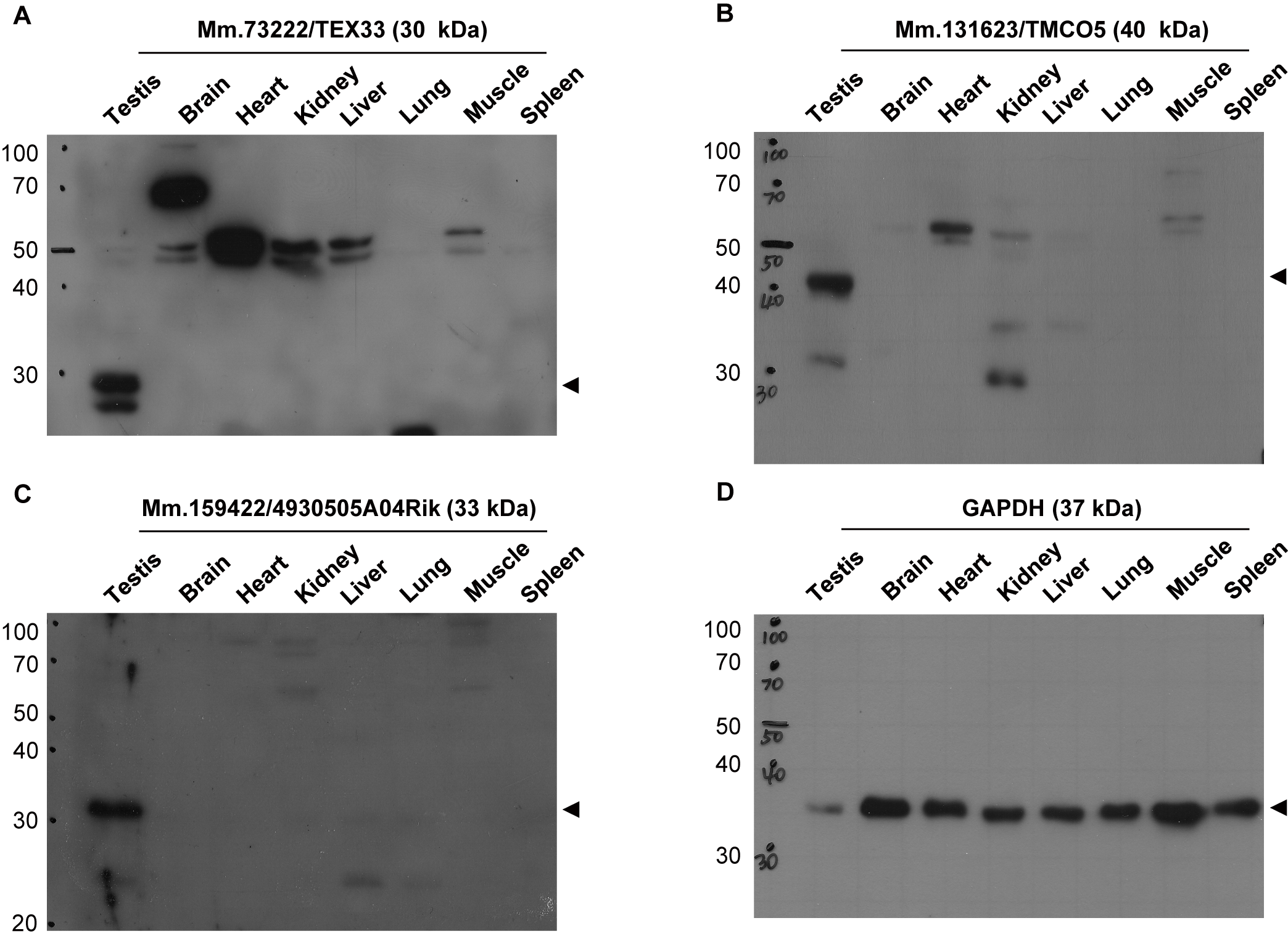

Supplement: S4 Fig — These are original uncropped and unadjusted blots of three proteins in Fig 4A. Bands corresponding to the proteins are indicated by arrowheads. (TIF) [file pone.0182038.s004.tif]

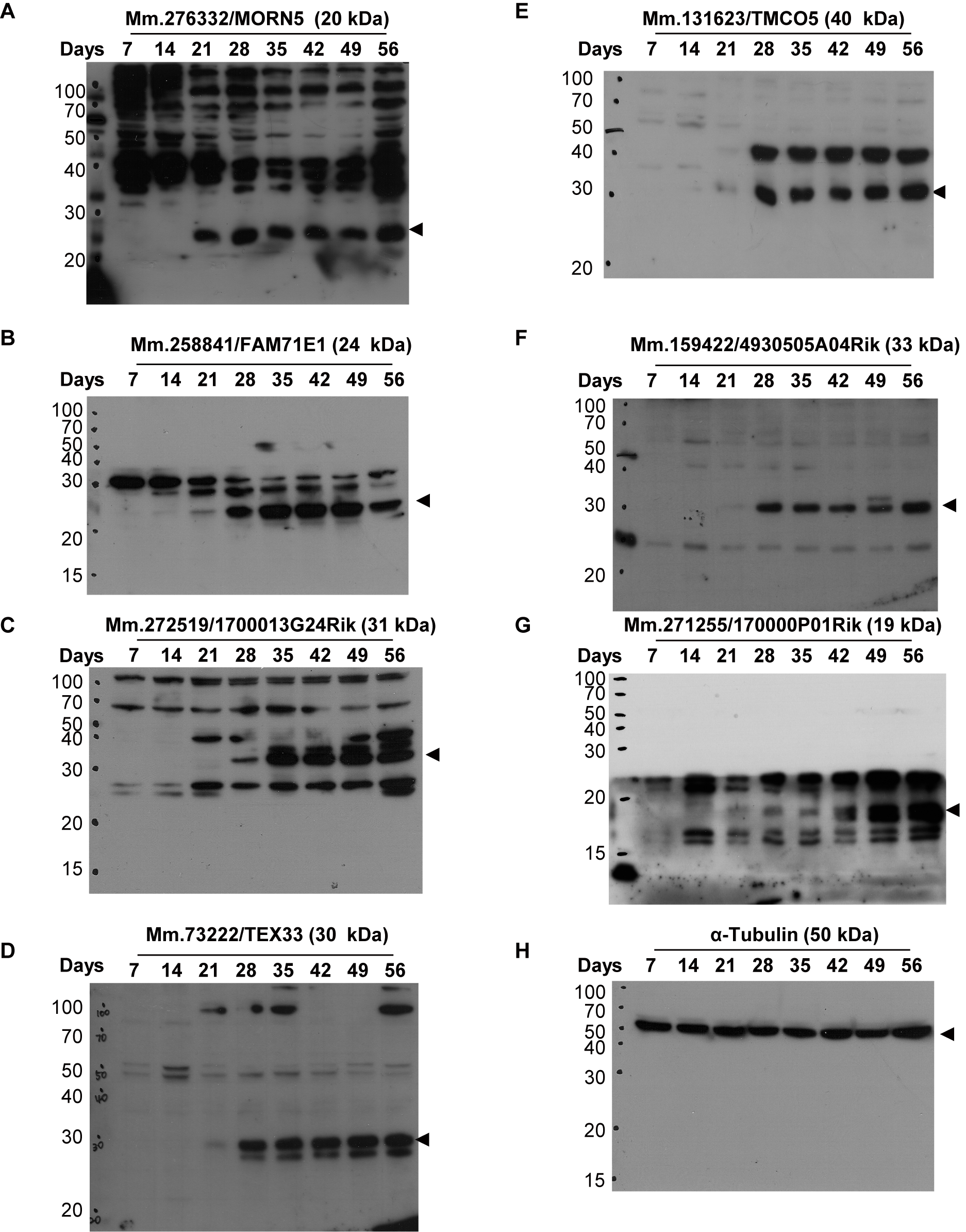

Supplement: S5 Fig — These are original uncropped and unadjusted blots of the proteins in Fig 4B. Bands corresponding to the proteins are indicated by arrowheads. (TIF) [file pone.0182038.s005.tif]

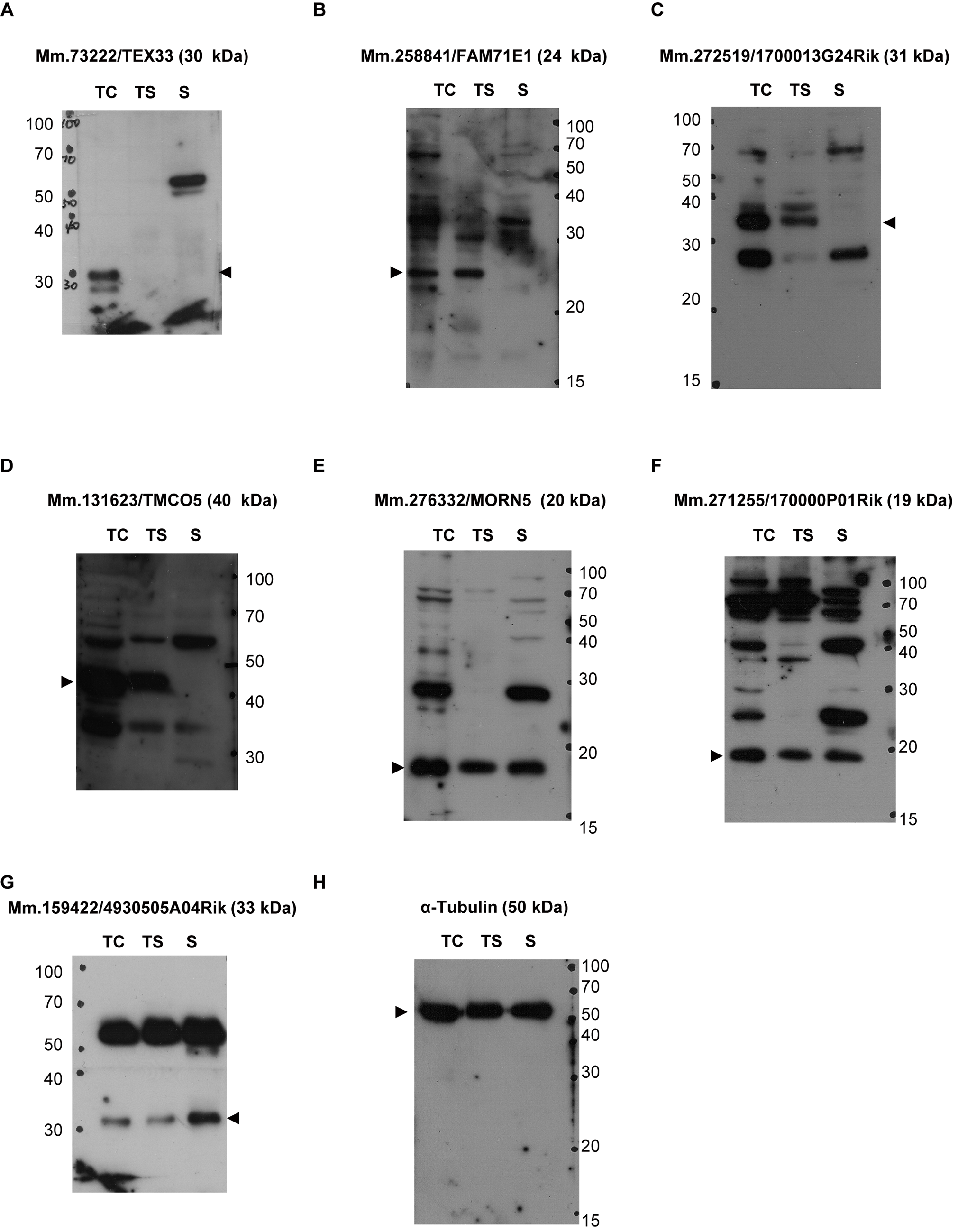

Supplement: S6 Fig — These are original uncropped and unadjusted blots of the proteins in Fig 5A. Bands corresponding to the proteins are indicated by arrowheads. (TIF) [file pone.0182038.s006.tif]

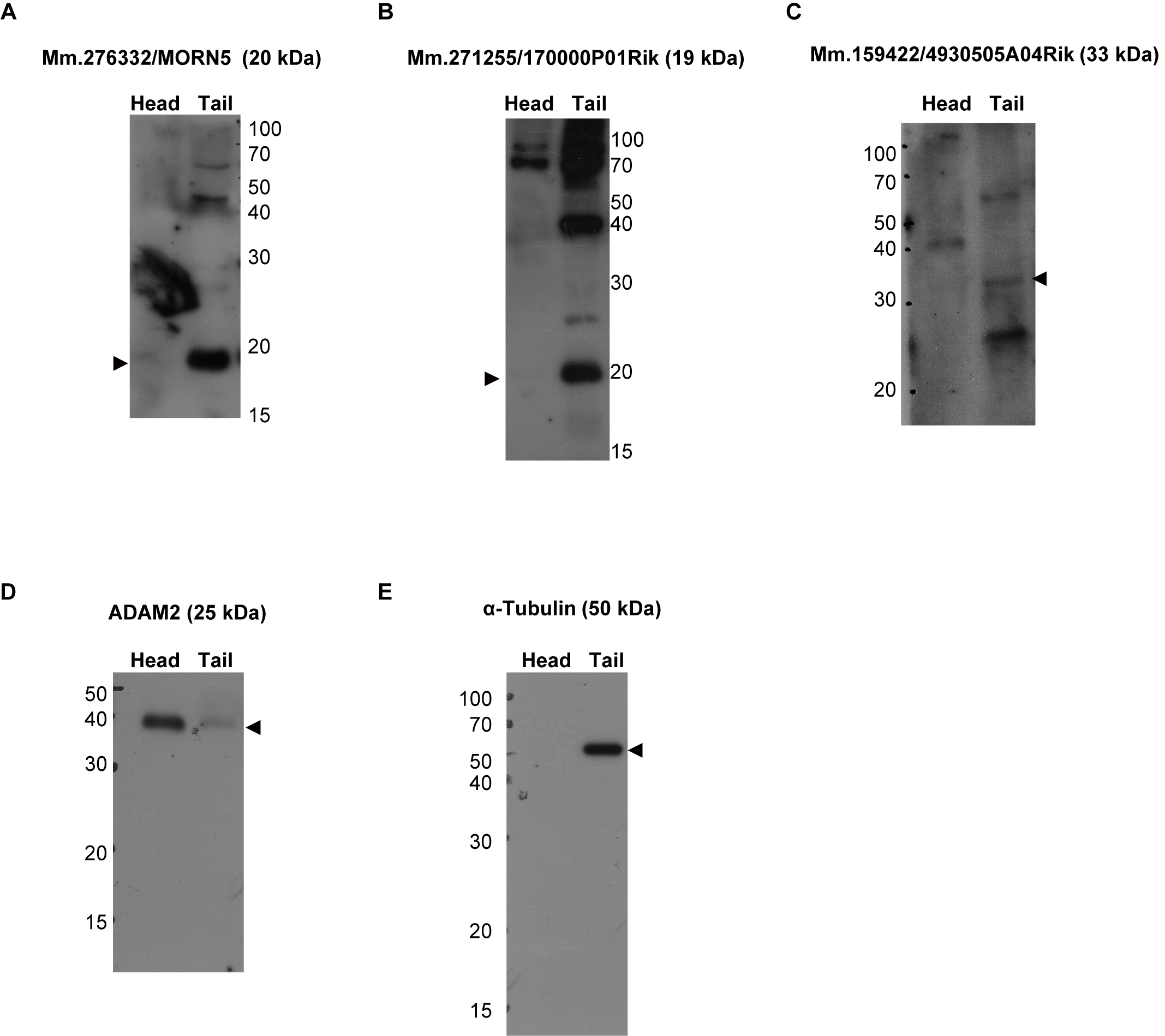

Supplement: S7 Fig — These are original uncropped and unadjusted blots of the proteins in Fig 5B. Bands corresponding to the proteins are indicated by arrowheads. (TIF) [file pone.0182038.s007.tif]

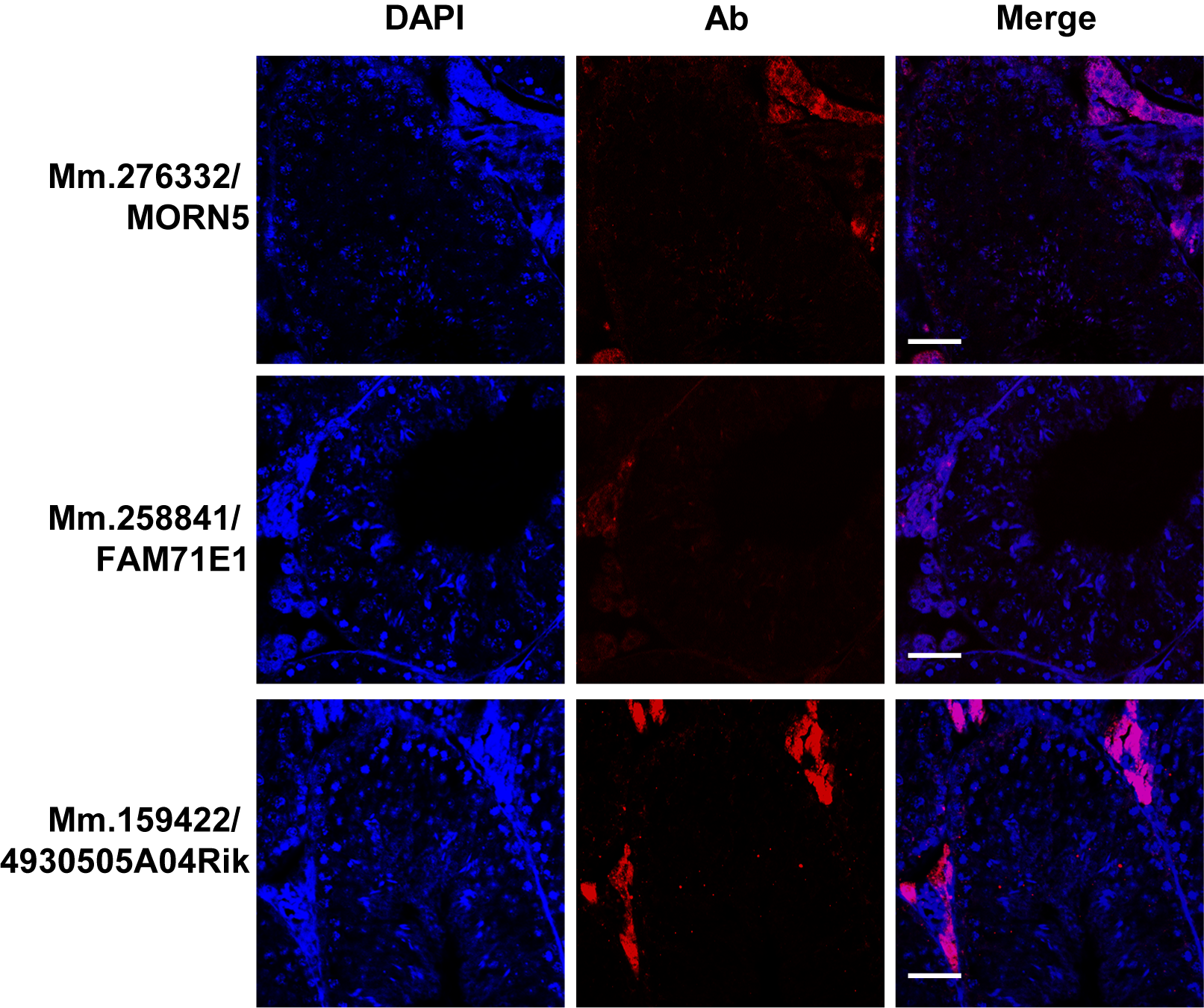

Supplement: S8 Fig — Immunofluorescence staining of paraffin sections of adult testis was conducted using specific antibodies to Mm.276332/MORN5, Mm.258841/FAM71E1, and Mm.159422/4930505A04Rik. Nuclei was stained with DAPI (blue). These antibodies did not display immunoreactivity. Scale bar, 100 μm. (TIF) [file pone.0182038.s008.tif]

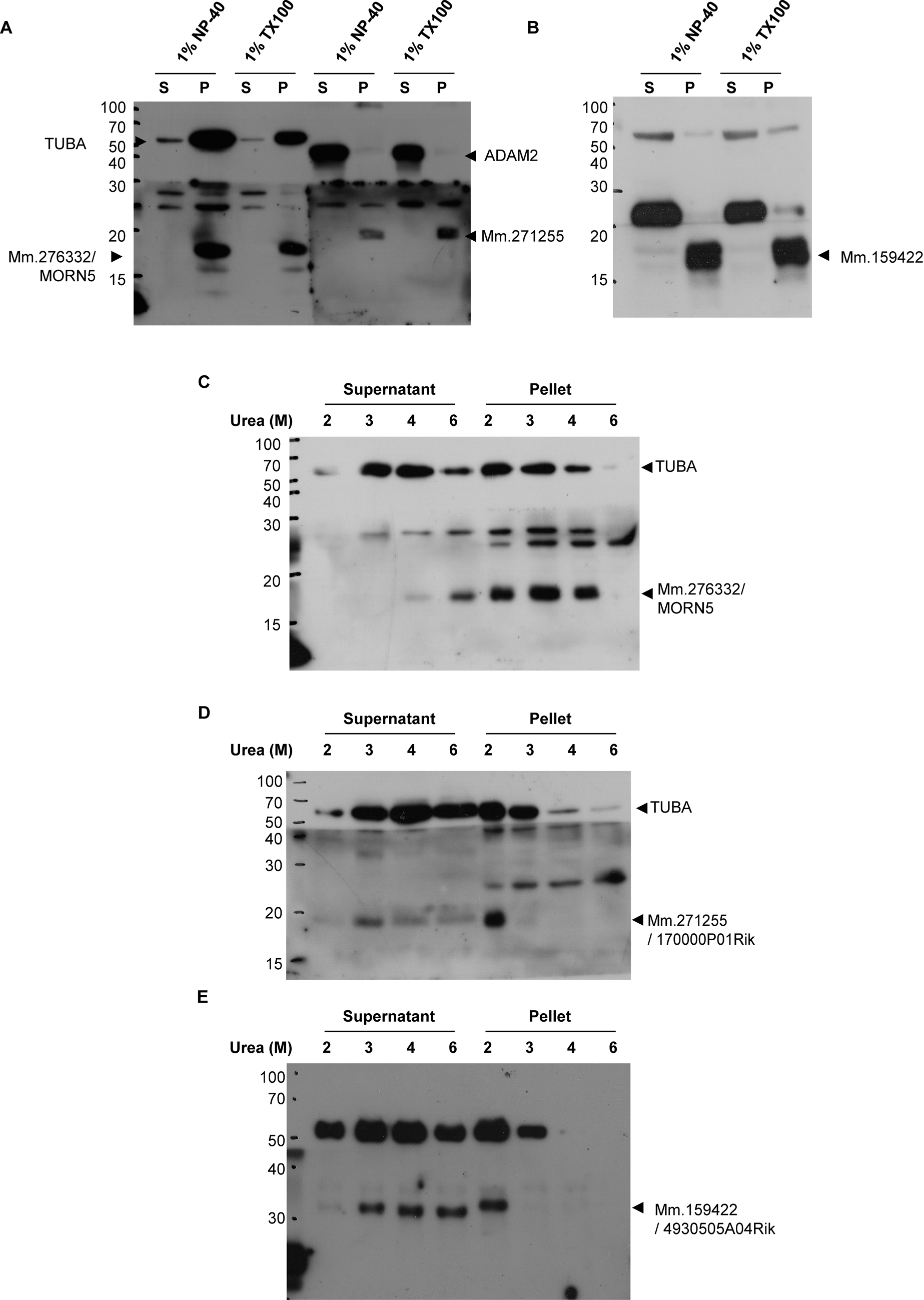

Supplement: S9 Fig — These are original uncropped and unadjusted blots of the proteins in Fig 7. Bands corresponding to the proteins are indicated by arrowheads. (TIF) [file pone.0182038.s009.tif]

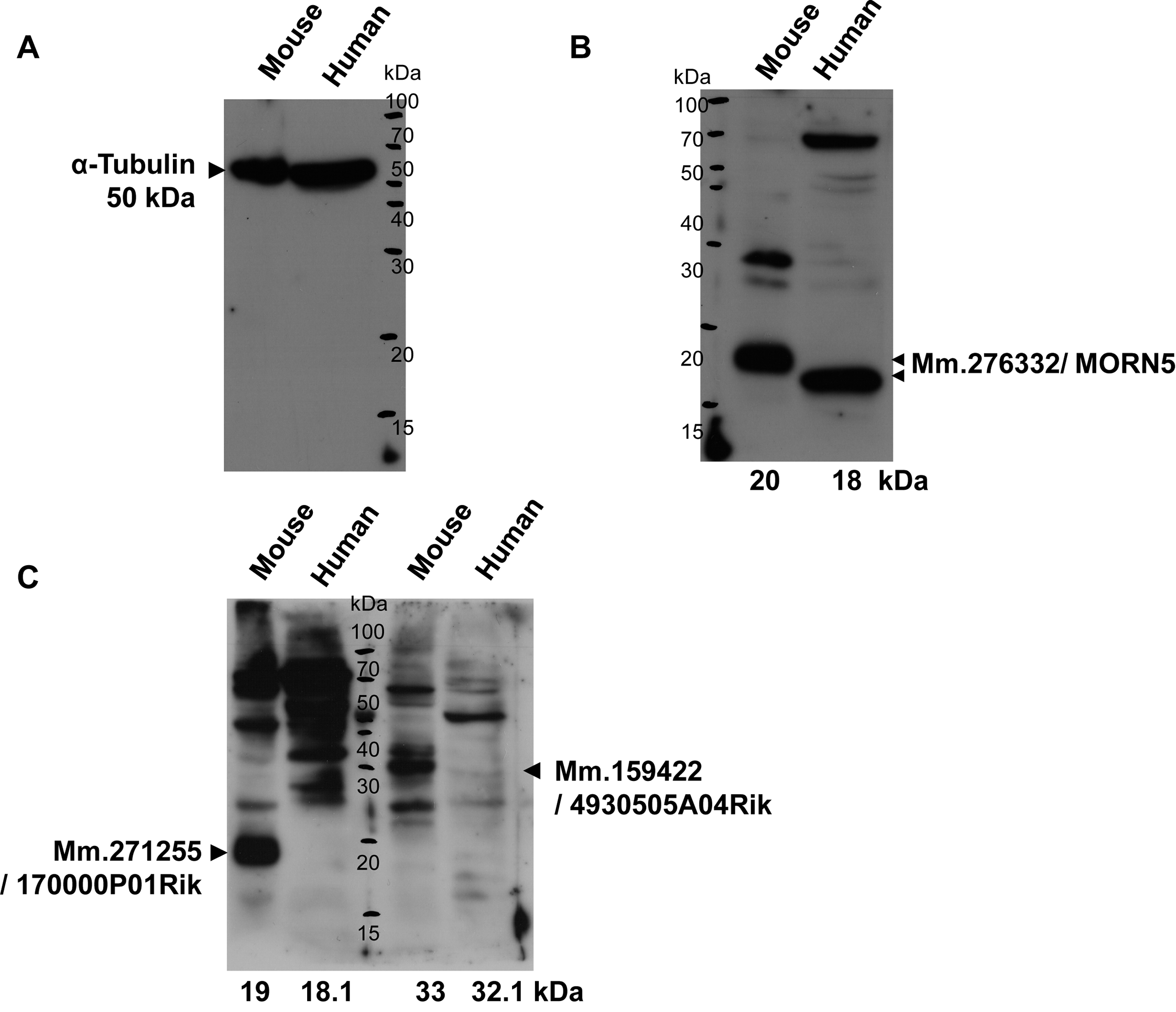

Supplement: S10 Fig — Extracts of sperm from mouse and humans were subjected to SDS-PAGE and blotted. Tubulin was detected as a loading control. MORN5 was expressed in human sperm (A). Mm.271255 (B) and Mm.159422 (C) antibodies did not cross-react with human orthologous proteins. Bands corresponding to the proteins are indicated by arrowheads. (TIF) [file pone.0182038.s010.tif]
