# Supplementary material for: Expression of uncharacterized male germ cell-specific genes and discovery of novel sperm-tail proteins in mice
Source: PLoS One. 2017 Jul 25;12(7):e0182038. doi: 10.1371/journal.pone.0182038 (PMC5526581; doi:10.1371/journal.pone.0182038)
Supplement: S1 Table — (DOCX) [file pone.0182038.s011.docx]

| UniGene ID | GeneBank ID | Purpose | PCR primers | | Size (bp) |
| --- | --- | --- | --- | --- | --- |
|  |  |  | Forward (5'-3') | Reverse (5'-3') |  |
| Mm.276332 | NM_029309 | RT-PCR | TGACGCCAAGACAGC | TCCCTGGTGAGATGC | 597 |
|  |  | Cloning | GGGATCCCCGACAAACACTGGCACTAC | CGCTCGAGTTTAGGACTTCTGTTTGGGC | 282 |
| Mm.23509 | NM_028560 | RT-PCR | CAGGAGAGAACAACG | ACCACCTAAGTCTGC | 638 |
| Mm.56430 | NM_025724 | RT-PCR | TGAAGAGGGAAGGCGTGGTC | GGAAAGGAGCGACGAGGGTG | 616 |
| Mm.73222 | NM_001163612 | RT-PCR | GGACACACCCGAACGACAAG | CTCCGTATTTCTCTTCCAGCG | 734 |
|  |  | Cloning | CGTGGATCCCCACAAGGACACACCCG | GCGAATTCCTCAGTCCTTCTTGCTCAG | 363 |
| Mm.131623 | NM_026104 | RT-PCR | GCCAAAGCACATCGGTAGAAG | CTTCCATTATCTTGCCGAGC | 528 |
|  |  | Cloning | CGTGGATCCCCAGATTGGAGAGGAAG | GCGAATTCCTCAACTCCTTTCTCTCTC | 369 |
| Mm.269049 | NM_028156 | RT-PCR | TGCGGTGGTTAGGATGGCTTG | GCCTTCGGGTTGGAGTTGTAG | 374 |
| Mm.157047 | NM_025750 | RT-PCR | CGTGGACATAGACCCTGAAC | CCTGTGGTTCTCCTACTGTTG | 664 |
| Mm.271255 | NM_029314 | RT-PCR | GCGACGAAGACAAAC | AGGTGGAGAAGAAGC | 465 |
|  |  | Cloning | CCGGAATTCCGAACCCTGCCTTACGC | CGACTCGAGCTACTTGGTTCCCTGG | 293 |
| Mm.272519 | NM_027063 | RT-PCR | GCCAACCCAACAACG | GAACTTGGGCAGACG | 749 |
|  |  | Cloning | GTGGGATCCCAAAGGGTCTGCGAAGCC | CCGGAATTCCTAGCGTTGACAGGTGGGC | 210 |
| Mm.87624 | NM_001289663 | RT-PCR | CTGTCCATCACCACCAAGG | AAGAGAAGTGCTGGTGCGG | 478 |
| Mm.258841 | NM_028169 | RT-PCR | GGCTGTTTCCCTTGC | TCTGGTCCTTTGGCG | 384 |
|  |  | Cloning | GTGGGATCCCCATGAAGGGCGGTCG | CCGGAATTCCTACTCGCCGCTCAG | 114 |
| Mm.46148 | NM_029608 | RT-PCR | CTGCTGAGATGGTGC | CTCTCTGGAACCTGC | 440 |
| Mm.159422 | NM_001100394 | RT-PCR | ATGGAGGGAGAGGAGAAGC | TTGGGGCTGTTCTGATG | 614 |
|  |  | Cloning | GGATCCTCGAGCCAGTTCCCTAC | GAATTCCGGCTGTGTTTGACTG | 191 |

**S1 Table.** Sequences of primers
