# Supplementary material for: Expression of uncharacterized male germ cell-specific genes and discovery of novel sperm-tail proteins in mice
Source: PLoS One. 2017 Jul 25;12(7):e0182038. doi: 10.1371/journal.pone.0182038 (PMC5526581; doi:10.1371/journal.pone.0182038)
Supplement: S2 Table — (DOCX) [file pone.0182038.s012.docx]

**S2 Table.** List of 111 proteins previously identified in mature sperm tail

| Gene | UniGene | Method | Pattern | Region ^*^/Solubility^1^ | KO | PMID |
| --- | --- | --- | --- | --- | --- | --- |
| Slc22a14 | Mm.335525 | IF | Tail | principle piece | Infertile | 27811987 |
| AMPK | Hs.43322 | IF | Head (e,p) and tail | whole tail | nd | 27678462 |
| Cofilin | Mm.329655 | IF | Tail | whole tail | nd | 27369112 |
| Ano5 | Mm.254431 | WB | Tail | whole tail | Subfertile | 26667038 |
| CAII/CAIV | Mm.1186/Mm.1641 | IF | Head (e,p) and tail | mid piece and principle piece | Subfertile | 26487715 |
| Tssk4 | Mm.171300 | IF | Tail | principle piece | Subfertile | 25361759 |
| Mfn2 | Mm.154312 | IF | Head (e,p) and tail | whole tail | nd | 24876927 |
| Ccr6 | Mm.8007 | IF | Head (p) and tail | whole tail | nd | 23765988 |
| Pmca4b | Mm.188617 | IF | Head and tail | principle piece | Infertile | 22020416, 15178683 |
| CASK | Mm.294882 | IF | Tail | principle piece | nd |  |
| Mns1 | Mm.387671 | IF | Tail | whole tail | Infertile | 22396656 |
| Lyzl4 | Mm.33351 | IF | Acrosome and tail | principle piece | nd | 21444326 |
| Trip11 | Mm.208618 | IF | Tail | whole tail | nd | 21337470 |
| Ift88 | Mm.4653 | IF | Acrosome and tail | whole tail | nd |  |
| Als2cr12 (SFAP3) | Mm.442063 | IF | Tail | whole tail | nd | 21402173 |
| Tekt5 | Mm.142181 | IF | Tail | mid piece | nd | 20378928 |
| Nssr1 | Mm.10229 | IF | Acrosome and tail | mid piece | nd | 21980524 |
| Znf645 | Hs.132485 | IF | Head (e,p) and tail | whole tail | nd | 20657603 |
| Spata18 | Rn.27773 | EM | Tail | mid piece | nd | 20108326 |
| Spef2 | Mm.127822 | IF | Acrosome and tail | mid piece | nd | 19889948 |
| Nsun4 | Mm.282560 | IF | Head and tail | whole tail | nd | 19686095 |
| Catsperg1 | Mm.349382 | IF | Tail | principle piece | nd | 19516020 |
| Cabs1 | Mm.272787 | IF | Acrosome and tail | principle piece | nd | 19208547 |
| Sept12 | Mm.87382 | IF | Head (e,p) and tail | mid piece | Infertile | 19359518 |
| Dnajb13 | Mm.278554 | IF | Tail | whole tail | nd | 19298648 |
| Vdac | Mm.3555 | IF | Head(p) and tail | whole tail | nd | 19013129 |
| SP-A | Hs.535295 | IF | Head(e) and tail | mid piece | nd | 18191856 |
| SP-D | Hs.253495 | IF | Acrosome and tail | whole tail | nd |  |
| Odf2 | Mm.330116 | IF | Tail | whole tail | Infertile | 18398819 |
| Rnf141 | Mm.96867 | IF | Acrosome and tail | whole tail | nd | 18823591 |
| Spin1 | Mm.188432 | IF | Tail | mid piece | nd | 18645677 |
| Ccdc63 (SFAP1) | Mm.386907 | WB | Tail | insoluble | nd | 18652659 |
| 4933424G06Rik (SFAP2) | Mm.157049 | WB | Tail | insoluble | nd |  |
| Tsks | Mm.84502 | IF | Tail | whole tail | nd | 18495105 |
| Tssk2 | Mm.310201 | IF | Tail | whole tail | nd |  |
| Ggn | Mm.389886 | IF | Tail | principle piece and end piece | nd | 18502891 |
| Crisp2 | Mm.1296 | IF | Acrosome and tail | principle piece | nd |  |
| Jakmip1 | Mm.85280 | IF | Acrosome and tail | mid piece | nd | 17668444 |
|  |  | WB | - | insoluble |  |  |
| Pcdp1 | Mm.297290 | IHC | Tail | whole tail | Infertile | 18039845 |
| Ptchd3 | Mm.61213 | IF | Tail | mid piece | nd | 17904097 |
| Fscb | Mm.189552 | IF | Tail | principle piece | nd | 17855365 |
| EPPIN | Hs.121084 | IF | Tail | whole tail | nd | 17567961 |
| CLU | Hs.436657 | IF | Tail |  | nd |  |
| LTF | Hs.529517 | IF | Tail |  | nd |  |
| HSPE1 | Hs.1197 | IF | Tail | mid piece and principle piece | nd | 17595329 |
| DNAJB1 | Hs.515210 | IF | Tail | mid piece and principle piece | nd |  |
| HSPA1A | Hs.274402 | IF | Head(e) and tail | whole tail | nd |  |
| Tsga2 | Mm.12743 | IF | Tail | whole tail | nd | 17451891 |
| SLC25A31 | Hs.149030 | IF | Tail | principle piece | nd | 17137571 |
| ALDOA | Hs.513490 | IF | Tail | principle piece | nd |  |
| PKM | Hs.534770 | IF | Tail | principle piece | nd |  |
| Tbata | Mm.30227 | IF | Tail | principle piece | nd | 17196196 |
| Dnajb1 | Mm.282092 | IF | Acrosome and tail | principle piece and end piece | nd | 16955402 |
|  |  | WB | - | soluble |  |  |
| Fkbp4 | Mm.12758 | IF | Acrosome and tail | mid piece | infertile | 17307907 |
| Catsper3 | Mm.159795 | IF | Tail | principle piece | infertile | 17227845 |
| Catsper4 | Mm.79072 | IF | Tail | principle piece | infertile |  |
| PASK | Hs.397891 | IF | Tail | mid piece | nd | 17595531 |
| Calm1 | Mm.285993 | IF | Acrosome and tail | principle piece | nd | 17460096 |
|  |  | WB | - | insoluble |  |  |
| CCDC65 | Hs.512805 | IF | Head(p) and tail | whole tail | nd | 17089017 |
| δ-, κ-, and μ-opioid receptors | Hs.2353/Hs.106795/Hs.372 | IF | Head(e) and tail | whole tail | nd | 16984994 |
| Ak1 | Mm.29189 | IF | Tail | mid piece and principle piece | nd | 16790685 |
| Ak2 | Mm.29460 | IF | Tail | mid piece | nd |  |
| NR3C2 | Hs.163924 | IF | Tail | whole tail | nd | 16964418 |
| Na,K-ATPase a1 | Hs.371889 | IF | Tail | whole tail | nd | 16861705 |
| Na,K-ATPase a4 | Hs.662219 | IF | Tail | mid piece | nd |  |
| Na,K-ATPase b1 | Hs.291196 | IF | Tail | mid piece and principle piece | nd |  |
| Na,K-ATPase b3 | Hs.477789 | IF | Tail | whole tail | nd |  |
| Catsper1 | Mm.87321 | IF | Tail | principle piece | infertile | 16625279, 16036917 |
| Catsper2 | Mm.384318 | IF | Tail | principle piece | Infertile | 16970151, 16036917 |
| Tekt4 | Mm.282330 | IF | Tail | mid piece and principle piece | nd | 16596631 |
|  |  | WB | - | insoluble |  |  |
| Tsga10 | Mm.332756 | IF | Acrosome and tail | whole tail | nd | 16777103 |
| GNA12 | Hs.487341 | IF | Tail | mid piece | nd | 16612612 |
| Musk | Mm.16148 | IF | Head(p) and tail | mid piece | nd | 16487930 |
| Spag5 | Mm.24250 | EM | Tail | mid piece and principle piece | fertile | 16211599, 11884588 |
| Pde1a | Mm.40678 | IF | Tail | whole tail | nd | 15901640 |
| Dnah8 | Mm.426017 | IF | Tail | principle piece and end piece | nd | 16054618 |
| Spef1 | Mm.252722 | EM | Tail | mid piece and principle piece | nd | 15979255 |
| Spam1 | Mm.4688 | IF | Tail | mid piece and principle piece | nd | 15457544 |
| AQP7 | Hs.455323 | IF | Tail | mid piece | nd | 15540792 |
| SPA17 | Hs.286233 | IF | Tail | mid piece and principle piece | nd | 15257753 |
| Tsga10 | Mm.332756 | IF | Head and tail | principle piece | nd | 14585816 |
| AKAP3 | Hs.98397 | IF | Tail | principle piece and end piece | nd | 14996943 |
| LEPR | Hs.723178 | IF | Tail | mid piece and principle piece | nd | 14636218 |
| LCN6 | Hs.522504 | IF | Head (e,p) and tail | whole tail | nd | 14617364 |
| Nme8 | Mm.279939 | IF | Tail | principle piece | nd | 12909633 |
| SPAG1 | Hs.591866 | IF | Head and tail | mid piece and principle piece | nd | 12846798 |
| Akap4 | Mm.1498 | IF | Tail | principle piece and end piece | Infertile | 12606363, 12167408 |
| ODF4 | Hs.186045 | IF | Tail | mid piece and principle piece | nd | 12728016 |
| Rnf38 | Mm.262859 | IF | Tail | whole tail | nd | 12533418 |
| Arc | Mm.491310 | IF | Acrosome and tail | principle piece and end piece | nd | 12493697 |
| Txndc2 | Mm.255732 | WB | Tail | whole tail | nd | 12390887 |
| Tex22 | Mm.23377 | IF | Tail | mid piece | nd | 12359214 |
| Spag6 | Mm.31701 | IF | Tail | whole tail | Infertile | 12167721 |
| CD46 | Hs.510402 | IF | Acrosome and tail | mid piece | nd | 12112588 |
| CAPZA3 | Hs.131288 | IF | Head(p) and tail | whole tail | nd | 12029070 |
| TEKT2 | Hs.127111 | IF | Head(e) and tail | whole tail | nd | 12029069 |
| Odf3 | Mm.56404 | IF | Tail | whole tail | nd | 11870087 |
| Gas8 | Mm.117265 | IF | Tail | whole tail | nd | 11751847 |
| Cd80 | Mm.89474 | ICT | Acrosome and tail | whole tail | nd | 11883748 |
| BC100451 | Mm.439815 | IF | Tail | principle piece | nd | 11420250 |
| Pmfbp1 | Mm.42166 | IHC | Tail | whole tail | nd | 11468771 |
| TXNDC2 | Hs.98712 | IF | Head (e,p) and tail | whole tail | nd | 11399755 |
| Klc3 | Mm.213403 | IF | Tail | whole tail | Infertile | 11319135, 22561200 |
| HSPA2 | Hs.432648 | IF | Tail | whole tail | nd | 10952940 |
| Stard10 | Mm.28896 | IF | Head(p) and tail | whole tail | nd | 10819773 |
| Eno1 | Mm.70666 | IF | Tail | whole tail | nd | 10727018 |
| Ropn1 | Mm.23515 | IF | Tail | principle piece and end piece | Infertile | 10591629, 23303679 |
| Spag4 | Mm.330713 | EM | Tail | mid piece and principle piece | Infertile | 10373309, 26417726 |
| DAZ2 | Hs.592257 | IF | Tail | whole tail | nd | 9557839 |
| Krt5 | Rn.129725 | IF | Tail | whole tail | nd | 8858608 |
| Odf1 | Rn.9850 | IF | Tail | mid piece and principle piece | Infertile | 7521678, 22037768 |

IF, immunofluorescence; WB, western blotting

*: Distribution of the protein in sperm tail

1: Solubility of the protein in non-ionic detergents
