# Supplementary material for: Expression of uncharacterized male germ cell-specific genes and discovery of novel sperm-tail proteins in mice
Source: PLoS One. 2017 Jul 25;12(7):e0182038. doi: 10.1371/journal.pone.0182038 (PMC5526581; doi:10.1371/journal.pone.0182038)
Supplement: S3 Table — (DOCX) [file pone.0182038.s013.docx]

**S3 Table.** Gene Ontology terms related to sperm tail proteins

| Category | Term | Count | % | *p*-value | Fold enrichment |
| --- | --- | --- | --- | --- | --- |
| GOTERM_BP_DIRECT | spermatogenesis | 18 | 16.1 | 1.80E-14 | 12.8 |
| GOTERM_BP_DIRECT | sperm motility | 11 | 9.8 | 2.50E-14 | 46.6 |
| GOTERM_BP_DIRECT | multicellular organism development | 16 | 14.3 | 1.20E-06 | 4.5 |
| GOTERM_BP_DIRECT | cell differentiation | 14 | 12.5 | 1.50E-06 | 5.2 |
| GOTERM_BP_DIRECT | sperm capacitation | 4 | 3.6 | 7.00E-05 | 48.7 |
| GOTERM_BP_DIRECT | membrane depolarization during action potential | 4 | 3.6 | 1.10E-04 | 41.8 |
| GOTERM_BP_DIRECT | cell projection organization | 6 | 5.4 | 1.50E-04 | 11.7 |
| GOTERM_BP_DIRECT | calcium ion transmembrane transport | 5 | 4.5 | 2.30E-04 | 16.4 |
| GOTERM_BP_DIRECT | sperm axoneme assembly | 3 | 2.7 | 9.90E-04 | 62.7 |
| GOTERM_BP_DIRECT | calcium ion transport | 5 | 4.5 | 1.30E-03 | 10.4 |
| GOTERM_BP_DIRECT | axoneme assembly | 3 | 2.7 | 2.00E-03 | 43.9 |
| GOTERM_BP_DIRECT | cilium movement | 3 | 2.7 | 5.20E-03 | 27.4 |
| GOTERM_BP_DIRECT | transmembrane transport | 6 | 5.4 | 7.40E-03 | 4.8 |
| GOTERM_CC_DIRECT | motile cilium | 21 | 18.8 | 1.80E-31 | 65.4 |
| GOTERM_CC_DIRECT | sperm flagellum | 14 | 12.5 | 4.60E-24 | 112.8 |
| GOTERM_CC_DIRECT | sperm principal piece | 11 | 9.8 | 9.50E-19 | 115.9 |
| GOTERM_CC_DIRECT | cell projection | 24 | 21.4 | 1.80E-16 | 9.3 |
| GOTERM_CC_DIRECT | cilium | 17 | 15.2 | 4.30E-16 | 18.7 |
| GOTERM_CC_DIRECT | outer dense fiber | 6 | 5.4 | 2.40E-10 | 149.4 |
| GOTERM_CC_DIRECT | CatSper complex | 5 | 4.5 | 1.90E-08 | 152.2 |
| GOTERM_CC_DIRECT | acrosomal vesicle | 8 | 7.1 | 1.40E-07 | 19.9 |
| GOTERM_CC_DIRECT | cytoskeleton | 18 | 16.1 | 2.90E-07 | 4.4 |
| GOTERM_CC_DIRECT | axoneme | 7 | 6.2 | 5.70E-07 | 22.8 |
| GOTERM_CC_DIRECT | sperm midpiece | 5 | 4.5 | 7.10E-07 | 68.5 |
| GOTERM_CC_DIRECT | microtubule | 10 | 8.9 | 3.10E-06 | 8.2 |
| GOTERM_CC_DIRECT | sperm fibrous sheath | 4 | 3.6 | 1.60E-05 | 78.3 |
| GOTERM_CC_DIRECT | cytoplasm | 42 | 37.5 | 2.80E-05 | 1.7 |
| GOTERM_CC_DIRECT | microtubule cytoskeleton | 6 | 5.4 | 2.60E-04 | 10.4 |
| GOTERM_CC_DIRECT | centriole | 5 | 4.5 | 7.10E-04 | 12.2 |
| GOTERM_CC_DIRECT | neuron projection | 8 | 7.1 | 7.80E-04 | 5.2 |
| GOTERM_MF_DIRECT | voltage-gated calcium channel activity | 4 | 3.6 | 3.40E-04 | 28.7 |
| GOTERM_MF_DIRECT | calcium activated cation channel activity | 3 | 2.7 | 8.00E-04 | 69.6 |
| GOTERM_MF_DIRECT | calcium channel activity | 4 | 3.6 | 2.80E-03 | 14 |
| GOTERM_MF_DIRECT | GTPase binding | 3 | 2.7 | 4.00E-03 | 31.2 |
| GOTERM_MF_DIRECT | microtubule binding | 5 | 4.5 | 4.20E-03 | 7.5 |
| GOTERM_MF_DIRECT | kinesin binding | 3 | 2.7 | 8.30E-03 | 21.5 |
| GOTERM_MF_DIRECT | voltage-gated ion channel activity | 4 | 3.6 | 9.30E-03 | 9.1 |
| BP, biological process; CC, cellular component; MF, molecular function | | | | | |
